# Profiling Plasma Biomarkers, Particularly pTau217 and pTau217/Aβ42, and Their Relation to Cognition in Memory Clinic Patients

**DOI:** 10.1111/jnc.70182

**Published:** 2025-08-11

**Authors:** Marco Bucci, Ove Almkvist, Marina Bluma, Nicholas J. Ashton, Irina Savitcheva, Konstantinos Chiotis, Guglielmo Di Molfetta, Kaj Blennow, Henrik Zetterberg, Agneta Nordberg

**Affiliations:** ^1^ Department of Neurobiology, Care Sciences and Society, Centre for Alzheimer Research, Division of Clinical Geriatrics Karolinska Institutet Stockholm Sweden; ^2^ Turku PET Centre Turku University Central Hospital and University of Turku Turku Finland; ^3^ Department of Psychiatry and Neurochemistry, Institute of Neuroscience and Physiology, Sahlgrenska Academy University of Gothenburg Mölndal Sweden; ^4^ Medical Radiation Physics and Nuclear Medicine Karolinska University Stockholm Sweden; ^5^ Department of Neurology Karolinska University Hospital Stockholm Sweden; ^6^ Clinical Neurochemistry Laboratory Sahlgrenska University Hospital Mölndal Sweden; ^7^ Department of Neurodegenerative Disease UCL Institute of Neurology Queen Square London UK; ^8^ UK Dementia Research Institute at UCL London UK; ^9^ Hong Kong Center for Neurodegenerative Diseases Hong Kong; ^10^ Wisconsin Alzheimer's Disease Research Center, University of Wisconsin School of Medicine and Public Health University of Wisconsin‐Madison Madison Wisconsin USA; ^11^ Theme Inflammation and Aging Karolinska University Hospital Stockholm Sweden

**Keywords:** Alzheimer's disease, amyloid beta, neuropsychological testing, plasma biomarkers, tau

## Abstract

Alzheimer's disease (AD) is characterized by brain protein depositions, impaired synaptic transmission, and progressive cognitive decline. This clinical study, conducted at the tertiary memory clinic of Karolinska University Hospital in Stockholm, evaluates plasma pTau217 (in comparison to other plasma biomarkers) as a non‐invasive marker for predicting brain amyloid load and cognitive impairment. Uniquely, it integrates plasma biomarkers with cognitive profiling and amyloid PET to assess diagnostic utility across disease stages in a real‐world memory clinic setting. A total of 122 patients underwent extensive clinical examinations, including CSF analysis (used here for clinical diagnosis only), CT/MRI, neuropsychological (NP) testing (*n* = 80) and blood biomarker measurements. Prior to PET imaging, 74 patients were diagnosed with MCI among other diagnoses (AD, other dementia, no dementia). Following PET, patients were reclassified into diagnostic groups: MCI Aβ− (*n* = 29), MCI Aβ+ (*n* = 19), AD (*n* = 51), other dementias (*n* = 11). ROC analysis evaluated the ability of plasma biomarkers to predict Aβ‐PET positivity. NP test *z*‐scores were reduced into principal components (PCs) using PCA. Plasma pTau217 and pTau217/Aβ42 ratio were elevated in Aβ+ patients compared to MCI Aβ‐patients. The ratio distinguished MCI Aβ+ from AD and, together with pTau217, showed the highest predictive value for Aβ positivity in the MCI group among the biomarkers analyzed (AUC 92.8% and 91.4%). Plasma pTau217/Aβ42 ratio was associated with principal component PC2 (“memory encoding and recall”) in MCI Aβ+ (*ρ* =0.64, p=0.01) and negatively correlated with RAVL retrieval (PC2) in the same group (*ρ* =−0.57 and −0.6, p=0.028 and 0.017, respectively). Additionally, pTau217 correlated with the “Information” *z*‐score (PC4) in both AD (*ρ* = −0.50, *p* = 0.005) and MCI Aβ+ (*ρ* = 0.53, *p* = 0.042). Plasma pTau217/Aβ42 might be a valuable predictor of brain amyloid pathology and a potential marker of domain‐specific cognitive impairment in AD.

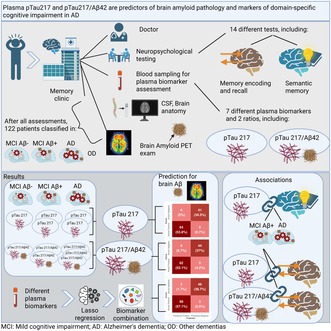

## Graphical abstract

This clinical study at Karolinska University Hospital evaluates plasma pTau217 and its ratio to Aβ42 as non‐invasive biomarkers for predicting brain amyloid pathology and cognitive impairment in Alzheimer's disease (AD). Among 122 patients, plasma pTau217/Aβ42 showed the highest predictive accuracy for amyloid PET positivity in mild cognitive impairment (MCI) cases (AUC 92.8%). Cognitive profiling revealed strong associations between this biomarker and memory‐related deficits. These findings suggest plasma pTau217/Aβ42 is a promising tool for early AD diagnosis and cognitive decline assessment in real‐world clinical settings.

**Abbreviations**
AD: Alzheimer's disease
APOE: apolipoprotein E
AUC: area under curve
Aβ: amyloid beta
CSF: cerebrospinal fluid
CT: computed tomography
FDG: fluorodeoxyglucose
FDR: false discovery rate
GFAP: Glial fibrillary acidic protein
InDaPCA: principal component analysis for incomplete data handling
LASSO: least absolute shrinkage and selection operator
LOOCV: cross‐validation with the leave‐one‐out method
MCI: mild cognitive impairment
MMSE: Mini‐Mental State Examination
MRI: magnetic resonance imaging
NFL: neurofilament light chain
NP: neuropsychological
NPV: negative predictive value
PC: principal component
PCA: principal component analysis
PET: positron emission tomography
PPV: positive predictive value
RAVLT: Rey auditory verbal learning test
ROC: receiver operating characteristic
ROCFT: Rey‐Osterrieth complex figure test
VR: visual reads

## 1 Introduction

Alzheimer's disease (AD) is a progressive neurodegenerative disorder that leads to memory loss and other cognitive problems. Early detection and monitoring of AD have improved considerably thanks to biomarkers targeting the pathognomonic for AD amyloid and tau pathologies. Among these, fluid biomarkers help us understand the key pathophysiological changes occurring in the brain due to AD; they are invaluable in both research settings, where they advance our knowledge of the disease, and clinical settings, where they assist in the diagnostic process (Zetterberg and Blennow 2021).

Recent technological advancements have enabled the measurement of these biomarkers in plasma, offering a less invasive and more accessible means of assessing AD pathology (Thijssen and Rabinovici 2021). The rapid progression in the development of plasma biomarkers has opened new avenues for early diagnosis and monitoring of disease progression (Ankeny et al. 2024). In support of these developments, recent studies have demonstrated strong correlations between plasma biomarkers and amyloid deposition in the brain, as measured by positron emission tomography (PET) scans, particularly in memory clinic cohorts (Dyer et al. 2024; Altomare et al. 2023; Bucci et al. 2023; Bluma et al. 2025).

Evaluating biomarkers, in their performance to predict amyloid in the brain and understanding the relationships between biomarkers and cognitive profiles, is crucial for reinforcing the validity of diagnosis and advancing the treatment of cognitive disorders. This can further validate the utility of plasma biomarkers in clinical and preclinical settings. Memory clinic‐based cohorts play a pivotal role in this effort, offering comprehensive assessments and often providing longitudinal data that can be used to predict disease progression.

Evidence regarding the prediction of amyloid pathology in the brain by plasma biomarkers has been accumulating, including the most promising biomarker pTau217 (Palmqvist et al. 2020; Ashton et al. 2024; Mattsson‐Carlgren et al. 2024). However, more studies, especially in heterogeneous memory clinic cohorts, are needed for testing/validating cut‐offs.

Initial evidence shows that plasma biomarkers associate with cognitive impairment measures, but researchers recognize that further research is needed to validate these findings in larger and more diverse cohorts (Xiao et al. 2021; Fernández Arias et al. 2025; Kim et al. 2025). Neuropsychological (NP) scores provide valuable insights into cognitive functions, but their interpretation can be complex due to numerous individual test scores that test overlapping cognitive domains. Plasma biomarker levels show correlations with different cognitive domains, such as memory, attention, and visuospatial function. Notably, pTau217 is particularly linked to memory. For instance, Xiao et al. (2021) found that higher levels of pTau217 are associated with poorer overall memory performance. Similarly, Fernández Arias et al. (2025) demonstrated that pTau217 levels correlate with specific aspects of memory, such as episodic and working memory, within the broader memory domain. Principal component analysis (PCA) is a statistical technique that can be used to reduce the dimensionality of complex data, in this case neuropsychological, by summarizing intercorrelated variables into a smaller set of uncorrelated components that aim to capture the most significant patterns in the data (Sperber 2021). These methods help to identify key cognitive domains in a data‐driven fashion and understand their variance across the different tests within neuropsychological batteries, like for example a research published on cognitively normal subjects (Hollinshead et al. 2022).

This study aimed to investigate the utility of plasma biomarkers, especially pTau217, as non‐invasive diagnostic tools for AD by predicting brain amyloid (Aβ) load and evaluating their relationship with cognitive impairment in a cohort of patients referred to a tertiary memory clinic due to memory problems. Unlike other clinical biomarker studies that apply inclusion and exclusion criteria, this work uniquely combines plasma biomarker data with detailed cognitive profiling and amyloid PET imaging in a real‐world clinical population. This approach enables stratification into diagnostic categories (e.g., MCI Aβ+, AD) and allows for the assessment of biomarker performance across different disease stages. The study also explores the added value of plasma pTau217 in refining clinical diagnoses and its potential role in identifying prodromal AD.

## 2 Methods

### 2.1 Participants

**FIGURE 1:**
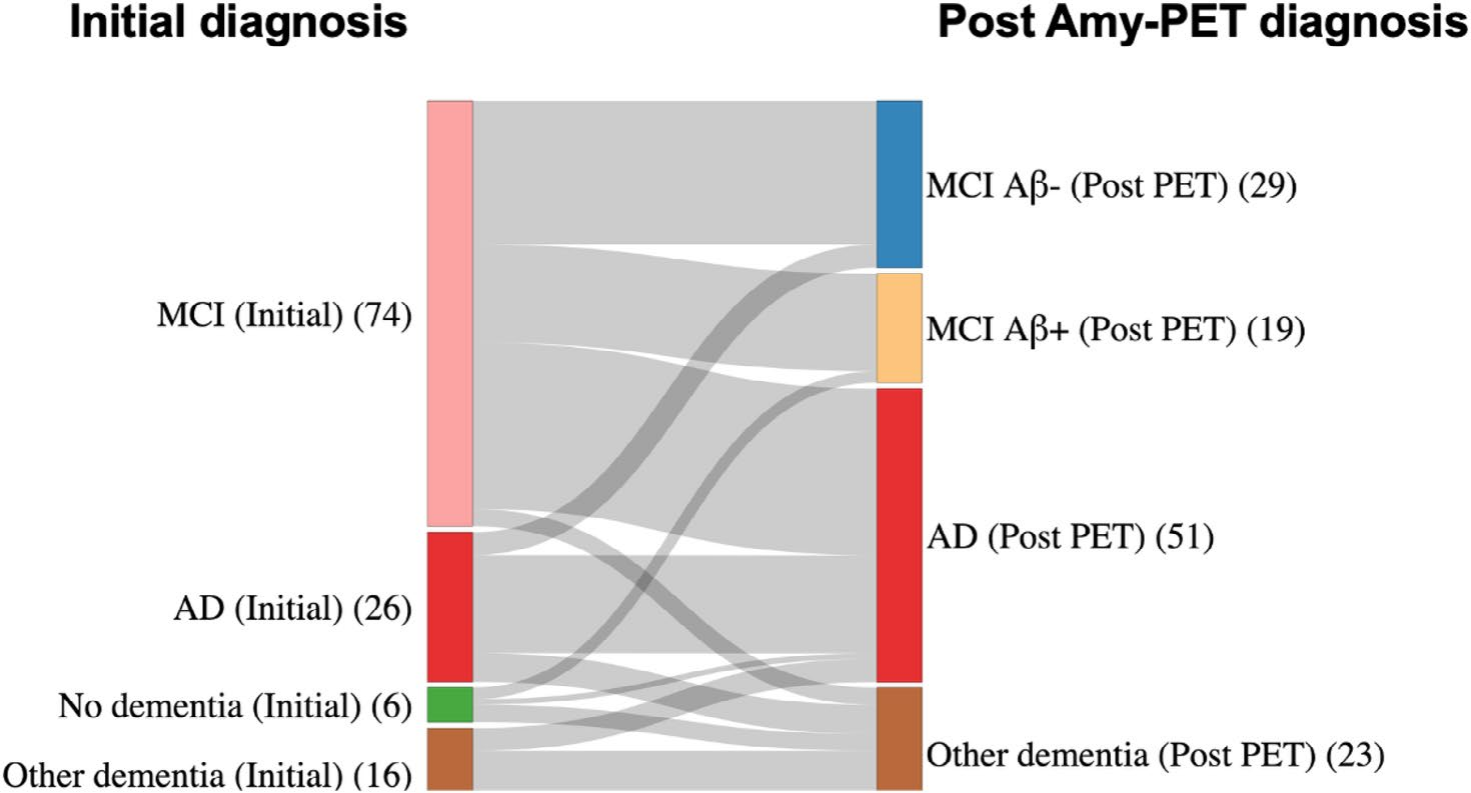
Diagnoses based on extensive assessment without considering PET evaluation (“Initial diagnosis”) and after Amyloid‐PET brain imaging scan has been acquired (“Post Amy‐PET diagnosis”).

This clinical study (not pre‐registered) included 122 patients from the Cognitive assessment unit at Karolinska University Hospital Huddinge, Stockholm, who underwent memory assessment. All subjects admitted to the clinic with impaired cognition and plasma samples and amyloid PET scanning available were included. Patients, mostly under 65, had been referred by general practitioners or other hospital clinics for cognitive problems. The patients underwent extensive assessments including medical history recording, often with a relative present, physical, neurological, psychiatric assessments, neuropsychological testing, cerebrospinal fluid sampling, CT or MRI, and for some cases PET‐FDG. An “initial diagnosis” followed and the classes were: No dementia (including subjective cognitive decline), MCI (Petersen 2004), AD (World Health Organization 1992), and “Other dementias” (including: Lewy body dementia, frontotemporal dementia, vascular dementia, and dementia not otherwise specified). For enhancing the diagnostic certainty of the “Initial diagnosis,” amyloid PET scans were performed at a relatively short interval after the initial memory assessments with less than 1.5 years between the initial visit (with neuropsychological testing and blood sampling) and PET imaging. The final diagnoses after PET (“Post Amy‐PET diagnosis”) had similar classes to initial diagnosis but the MCI class could be separated in MCI Aβ− and MCI Aβ+ (Prodromal AD). This diagnostic approach is consistent with the recent International Working Group (IWG) recommendations, which define Alzheimer's disease as a clinical‐biological construct—requiring a characteristic clinical phenotype supported by biomarker evidence such as amyloid PET, rather than relying solely on biological markers (Dubois et al. 2024). While both MCI Aβ+ and AD may fall under the biological definition of Alzheimer's disease, they are clinically distinguished based on severity through cognitive assessments conducted at Karolinska University Hospital, where the present study was carried out. MCI Aβ+ represents an earlier, prodromal stage with preserved daily functioning, whereas AD reflects a more advanced stage characterized by functional impairment in daily living. This diagnostic separation enables a more nuanced analysis of disease progression and biomarker relationships across stages. The change in diagnoses for each group are displayed in Figure 1. Of note, the 23 subjects included in the “Other dementias” group (Post Amy‐PET diagnosis) comprised: nine subcortical vascular dementia, three frontotemporal dementia, four Lewy body dementia (one of which was atypical PCA type), one alcohol related dementia, five unspecified dementia and even if not completely fitting in the category 1 PART (Primary Age‐Related Tauopathy). The study was conducted according to the guidelines of the Declaration of Helsinki. The study protocol was approved by the Ethics review authority of Sweden (2016‐120‐32; 2023‐05898‐02). Informed written consent was obtained from all participants in the study.

### 2.2 Data Acquisition

#### 2.2.1 Plasma Biomarker Analysis

During the clinical routine memory assessment, in conjunction with CSF sampling, approximately five tubes of blood were collected into sodium‐heparin tubes (Vacutainer, BD Diagnostics) and centrifuged (1500*g*, +4°C) for 10 min. Following centrifugation, the samples were aliquoted into polypropylene tubes and stored at −80°C within 30–60 min of collection. Pseudoanonymised samples (1 mL of plasma per subject) were sent to the Sahlgrenska hospital (University of Gothenburg) by temperature‐regulated dry ice transport. Sample analyses were conducted in a blinded manner with respect to the diagnosis. Aβ40, Aβ42, GFAP, and NFL concentrations were quantified using a multiplexed Single molecule array (Simoa, N4PE from Quanterix, Catalog number #103670) method. Plasma pTau217 was assessed with the ALZpath method (Catalog number #104570) (Ashton et al. 2024). Plasma pTau181 and pTau231 concentrations were measured using in‐house developed Simoa assays described previously (Karikari et al. 2020; Ashton et al. 2021).

#### 2.2.2 Neuropsychological Assessments

Eighty patients out of 122 had completed a full or partial battery of neuropsychological tests addressing different cognitive domains (Garcia‐Ptacek et al. 2014) including MMSE, as well as components of the Wechsler Adult Intelligence Scale (Coding, Symbol Search, Digit Span Forward and Backward, Arithmetic, Matrix Reasoning, Block Design, Information), Rey Auditory Verbal Learning Test (RAVLT), copying and memory subtests of the Rey‐Osterrieth Complex Figure Test (ROCFT), parts A and B of the Trail Making Test. The neuropsychological test results were transformed into *z*‐scores using a reference group of healthy subjects with comparable age, sex, and education to our cohort (Lezak et al. 2004).

Eight age‐matched cognitive healthy controls with MRI, NP testing, and plasma biomarker analysis were included from a clinical research project aimed at investigating autosomal dominant AD with different biomarkers and including healthy subjects with MMSE ≥ 28.

The dataset comprising the 88 subjects with neuropsychological assessments available is reported in Table S1.

#### 2.2.3 Amyloid PET Imaging

[18F]‐Flutemetamol PET scans were performed using either a Biograph mCT PET/CT scanner (Siemens/CTI, Knoxville, TN) or a GE Discovery scanner (General Electric, USA) at the Department of Medical Radiation Physics and Nuclear Medicine Imaging, Karolinska University Hospital, Huddinge, Sweden, according to the earlier description (Bluma et al. 2025).

In brief, the amyloid PET [18F]Flutemetamol scan was performed as a 20 min list‐mode scan 90 min after injection of 185 MBq [18F]‐Flutemetamol.

[18F]‐Flutemetamol summation images were visually assessed as positive or negative by an experienced nuclear medicine physician (I.S.) according to product‐specific guidelines. Additionally, PET images were pre‐processed using the robust PET‐only pipeline (rPOP; Iaccarino et al. 2022) in MATLAB (MathWorks, v.R2022a) and SPM 12. SUVr were first calculated using the whole cerebellum as the reference region, then Centiloids were calculated using an in‐house Centiloid calibration pipeline based on methods described by Klunk et al. (2015) and Battle et al. (2018).

### 2.3 Statistical Analysis—Calculations

Statistical analysis was carried out in R (version 4.4.2, https://www.r‐project.org), while data visualizations were created using ggplot2 (v3.5.1). Assessment for data normality and outlier inspection was performed. It was chosen to perform non‐parametric testing for all variables to not violate assumptions of parametric testing for those not normally distributed. Non‐parametric testing is also less affected by outliers, which were present in a few variables. For the PCA analysis (described in 2.3.2) the outliers were inspected with Multidimensional Scaling (cmdscale and dist from the basic R‐library: stats).

The boxplots are described in the Supporting Information.

The ratio between plasma pTau217 and plasma Aβ42 has been computed as previously done (Lehmann et al. 2025). Plasma biomarkers have been compared across diagnostic groups (“Post Amy‐PET diagnosis”) and if Kruskal–Wallis test across groups resulted significant, post hoc analysis with Tukey test with and without False Discovery Rate (FDR) correction for multiple comparisons have been performed and reported.

#### 2.3.1 ROC‐AUC

Receiver Operating Characteristic (ROC) analysis to predict for Aβ positivity (Visual Read, VR) was performed on the whole clinical cohort (*n* = 122) and separately on the MCI cohort (deriving from “Initial diagnosis” grouping) (*n* = 73). The plasma biomarkers were corrected for age and sex effects in each group to avoid overadjustment bias (Gao et al. 2025) and the ROC analysis was performed with the pROC library using Youden's index as the criterion of maximization. ROC analysis was performed with both individual plasma biomarker levels and with a combination of multiple plasma biomarkers selected with Lasso regression. Lasso regression was used on all plasma biomarkers (please refer to Figure 3 and Table 2) to create a ROC predictive model for Aβ positivity. This process identified only the significant biomarkers. The model was then tested using nested cross‐validation with the leave‐one‐out method (LOOCV) to make the most of the available data. The best lambda (Lasso parameter) was selected from the cross‐validation fold with the smallest Normalized Root Mean Square Error. To enhance the clinical interpretability of our findings, we calculated positive and negative predictive values (PPV and NPV) for key tests using both the observed prevalence in our cohort (58% in the full sample; 64% in the MCI subgroup diagnosed after initial assessment) and prevalence estimates from the literature (30% for SCD, 50% for MCI, and 65% for dementia), as reported in Meyer et al. (2024). To address the potential risks of overfitting, particularly given the modest sample size and lack of external validation, we conducted additional analyses to evaluate model calibration (calibration plots) and overall predictive accuracy (Brier score).

#### 2.3.2 Principal Component Analysis

*InDaPCA* (Principal Component Analysis for Incomplete Data handling)—PCA is a reduction technique that allows reducing the number of variables, in this case the NP tests, to a smaller number of principal components. *InDaPCA* is a regularized iterative PCA that can handle missing data without pre‐imputation, managing up to 58% missing values for a single variable (Podani et al. 2021). It was recently used in a neuroimaging study (Kumar et al. 2025), and PCA has previously been applied to reduce neuropsychological dimensions in dementia (Rivera‐Fernández et al. 2021).

In our study, the principal components (PCs) were retained based on the Keiser Criterion (Braeken and van Assen 2017). To identify the major test contributors for each PC, we used a supervised feature selection approach (Rahmat et al. 2024). More details on the PCA computation methods are available in the Supporting Information and Methods section. Both NP variables and plasma biomarkers were adjusted for their linear associations with covariates. NP variables were adjusted for age, sex, and education, while plasma biomarkers were adjusted for age and sex. This involved extracting residuals from regression models and re‐evaluating adjustments for each subgroup to ensure optimal correction, excluding non‐significant covariates (Pourhoseingholi et al. 2012). Missing values were handled in a pairwise fashion to maximize the usage of data for the correlations and other analyses.

Due to the non‐normal distribution of some variables, the relationships between plasma biomarkers and amyloid load (Centiloid) and the principal components derived by PCA and neuropsychological test *z*‐scores were tested using the Spearman's rank correlation coefficient.

Significant results were reported as *p* < 0.05 but, for regression analyses, given the number of tests performed and the relatively small sample size, *p* < 0.01 was considered a good threshold for significance. When appropriate, and mentioned in the text, False Discovery Rate (FDR) multiple comparison correction has been performed.

## 3 Results

### 3.1 Description of the Clinical Cohort (Post Amy‐PET Diagnosis)

Table 1 presents the general characteristics of the clinical cohort, including demographic, clinical, and biomarker data across diagnostic groups. There were no significant differences in age, sex, education, or MMSE scores among the groups, as assessed by the Kruskal–Wallis test (*p* > 0.05).

**TABLE 1:** General characteristics of the clinical cohort.

Variable	MCI Aβ− *N* = 29	MCI Aβ+ *N* = 19	AD *N* = 51	Other dementias *N* = 23	All *N* = 122	*p* *
Age	—	—	—	—		0.713^1^
Mean (SD)	66 (11)	67 (8)	64 (7)	66 (9)	65 (9)	
Min, Max	44, 86	54, 80	48, 83	42, 81	42, 86	
Sex	—	—	—	—		0.081^2^
F	16 (55%)	15 (79%)	28 (55%)	9 (39%)	68 (56%)	
M	13 (45%)	4 (21%)	23 (45%)	14 (61%)	54 (44%)	
Education	—	—	—	—		0.445^1^
Mean (SD)	12.5 (2.3)	13.3 (3.9)	12.7 (3.1)	11.6 (3.7)	12.6 (3.2)	
Min, Max	9.0, 17.0	7.0, 20.0	5.0, 17.0	7.0, 21.0	5.0, 21.0	
Missing	7	2	13	9	31	
MMSE		d		b		0.005^1^
Mean (SD)	25.6 (3.4)	27.4 (1.9)	25.4 (3.4)	23.3 (4.0)	25.4 (3.5)	
Min, Max	17.0, 30.0	24.0, 30.0	17.0, 30.0	15.0, 29.0	15.0, 30.0	
Missing	0	0	1	1	2	
Centiloid	bc	ad	ad	bc		< 0.001^1^
Mean (SD)	−2 (14)	78 (25)	96 (27)	−1 (19)	52 (52)	
Min, Max	−23, 38	33, 121	38, 162	−38, 43	−38, 162	
Missing	2	0	1	0	3	
Amyloid beta positivity (VR)	bc	ad	ad	bc		< 0.001^2^
Aβ PET−	29 (100%)	1 (5.3%)	0 (0%)	22 (96%)	52 (43%)	
Aβ PET+	0 (0%)	18 (95%)	51 (100%)	1 (4.3%)	70 (57%)	
Initial diagnosis	cd	cd	abd	abc		< 0.001^3^
AD	4 (14%)	0 (0%)	17 (33%)	5 (22%)	26 (21%)	
MCI	25 (86%)	17 (89%)	29 (57%)	3 (13%)	74 (61%)	
No dementia	0 (0%)	2 (11%)	1 (2.0%)	3 (13%)	6 (4.9%)	
Other dementias	0 (0%)	0 (0%)	4 (7.8%)	12 (52%)	16 (13%)	

*Note:* CLD letters (a, b, c, d) indicate significant differences between groups. Letters a, b, c, d indicate, respectively, differences with MCI Aβ−, MCI Aβ+, AD, and Other dementias group.

Abbreviation: VR = visual read.

* ^1^Kruskal–Wallis test, ^2^Chi‐squared test, ^3^Fisher's Exact test.

### 3.2 Plasma Biomarkers by Diagnostic Groups (Post Amy‐PET Diagnosis)

**FIGURE 2:**
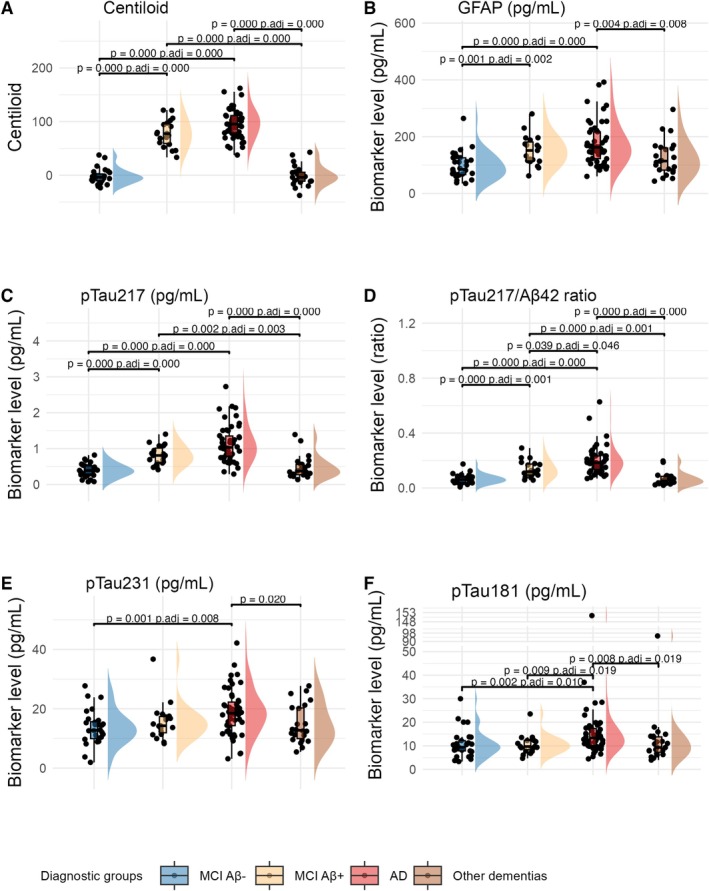
Brain amyloid deposition (Centiloid) and plasma biomarker levels (pg/mL) of pTau217, pTau231, pTau181, GFAP, and ratio pTau217/Aβ42 in different diagnostic groups (Post Amy‐PET diagnosis: MCI Aβ−, MCI Aβ+, AD, “Other dementias”). Only significant (*p* < 0.05) *p* values and *p* values adjusted for multiple comparisons (FDR) are reported.

Biomarker data by diagnostic group are presented in Table S2. Figure 2 illustrates brain amyloid load and fluid biomarker levels in the full cohort (*n* = 122).

Plasma pTau217 and its ratio with Aβ42 showed the greatest number of significant group differences. Specifically, plasma pTau217 levels were significantly lower in MCI Aβ− compared to both MCI Aβ+ and AD groups. The “Other dementias” group also exhibited lower pTau217 levels than both MCI Aβ− and AD. Additionally, plasma pTau217/Aβ42 levels differed significantly between MCI Aβ+ and AD. For plasma GFAP, three group comparisons survived FDR correction: levels were lower in MCI Aβ− compared to MCI Aβ+ and AD, and also lower in the “Other dementias” group compared to MCI Aβ+ and AD. These results highlight plasma pTau217 (especially) and GFAP as particularly sensitive biomarkers for distinguishing between early and advanced stages of neurodegenerative disease.

### 3.3 Plasma Biomarkers Correlations With Amyloid Load (Post Amy‐PET Diagnosis)

In the MCI group (*n* = 46), plasma pTau217 levels (pg/mL) showed a strong positive correlation with brain amyloid burden measured by PET Centiloid values (*ρ* = 0.74, *p* < 0.001). This association remained significant within the MCI Aβ+ subgroup (*n* = 19), though slightly attenuated (*ρ* = 0.60, *p* < 0.001). Additionally, MCI Aβ+ patients exhibited significantly higher amyloid load compared to MCI Aβ– patients based on visual PET reads (*p* < 0.001).

### 3.4 Plasma Biomarker‐Based Prediction of Amyloid PET (Initial Diagnosis)

#### 3.4.1 Biomarker Performance

**FIGURE 3:**
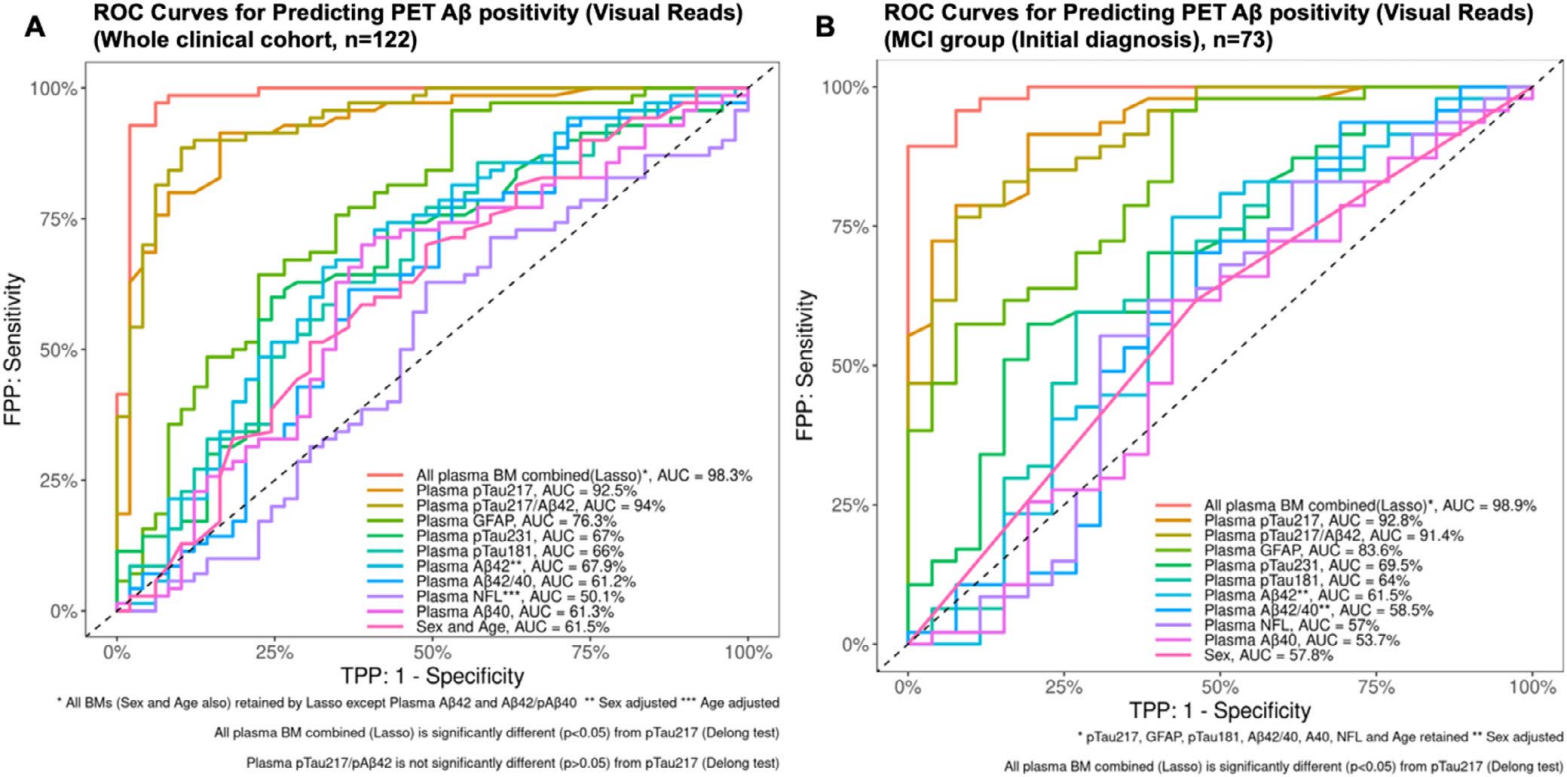
ROC curves of plasma biomarkers and the Lasso combination to predict PET Aβ positivity (VR) in the whole cohort (A) and in the MCI (before PET) (B) groups. Plasma pTau217 and plasma pTau217/Aβ2 ratio are the best individual biomarkers in predicting Aβ positivity for the whole cohort (panel A) and in the MCI group (Initial diagnosis, panel B). The combination of plasma biomarkers with Lasso regression was significantly better in prediction than any single biomarker or ratio.

ROC analyses (Figure 3, Table 2) revealed that plasma pTau217 was the most accurate individual biomarker for predicting Aβ pathology (VR‐PET) in the full clinical cohort, with an AUC of 92.5%, sensitivity of 91.4%, and PPV of 89%. Plasma pTau217/Aβ42 showed a slightly higher AUC (94%) and PPV (93%), but lower sensitivity (88.6%), and the difference in AUC was not statistically significant. A LASSO regression combining all biomarkers (excluding plasma Aβ42 and Aβ42/40) achieved an AUC of 98.3%, with a PPV of 96% and sensitivity of 97.1%, indicating the strong additive value of multi‐marker models.

**TABLE 2:** Results of ROC analyses of individual plasma biomarkers and the Lasso combination to predict PET Aβ positivity (VR) in the whole cohort and in the MCI (Initial diagnosis) groups.

Whole clinical cohort, *n* = 122—Prediction of amyloid PET positivity (VR)											
	AUC (%)	95% CI	Cut‐off	Specificity (%)	Sensitivity (%)	PPV	NPV	TN	TP	FN	FP
All plasma BM combined (Lasso)*	98.3	95.8–100	0.53	93.9	97.1	96	96	46	68	2	3
Plasma pTau217	92.5	87.7–97.4	0.58	83.7	91.4	89	87	41	64	6	8
Plasma pTau217/Aβ42	94	89.8–98.1	0.09	89.8	88.6	93	85	44	62	8	5
Plasma GFAP	76.3	67.4–85.3	86.37	46.9	95.7	72	88	23	67	3	26
Plasma pTau231	67	57–77	16.14	75.5	60	78	57	37	42	28	12
Plasma pTau181	66	55.9–76.1	9.51	53.1	75.7	70	60	26	53	17	23
Plasma Aβ42**	67.9	57.8–78	0.19	67.3	65.7	74	58	33	46	24	16
Plasma Aβ42/40	61.2	50.4–71.9	0.06	46.9	78.6	68	61	23	55	15	26
Plasma NFL***	50.1	39.2–61	−3.68	51	62.9	65	49	25	44	26	24
Plasma Aβ40	61.3	50.7–71.9	117.87	61.2	70	72	59	30	49	21	19
Sex and age	61.5	50.9–72	0.54	51	70	67	54	25	49	21	24

MCI (initial diagnosis), *n* = 73—Prediction of amyloid PET positivity (VR)											
	AUC (%)	95% CI	Cut‐off	Specificity (%)	Sensitivity (%)	PPV	NPV	TN	TP	FN	FP
All plasma BM combined (Lasso)*	98.9	97.3–100	0.65	100	89.4	100	84	26	42	5	0
Plasma pTau217	92.8	87.2–98.5	0.58	80.8	91.5	90	84	21	43	4	5
Plasma pTau217/Aβ42	91.4	85–97.8	0.11	92.3	76.6	95	69	24	36	11	2
Plasma GFAP	83.6	74.3–93	87.68	57.7	95.7	80	88	15	45	2	11
Plasma pTau231	69.5	56.7–82.3	15.39	80.8	57.4	84	51	21	27	20	5
Plasma pTau181	64	49.8–78.2	10.87	73.1	59.6	80	50	19	28	19	7
Plasma Aβ42**	61.5	46.7–76.4	0.79	57.7	76.6	77	58	15	36	11	11
Plasma Aβ42/40**	58.5	43.4–73.6	0.01	30.8	93.6	71	73	8	44	3	18
Plasma NFL	57	42–72.1	20.31	69.2	55.3	76	46	18	26	21	8
Plasma Aβ40	53.7	38.9–68.4	116.78	57.7	61.7	72	45	15	29	18	11
Sex	57.8	45.7–69.8	0.64	53.8	61.7	71	44	14	29	18	12

*Note:* In the whole cohort (top): *all plasma biomarkers (BM) include the full list of biomarkers below except plasma Aβ42 and plasma Aβ42/40 ratio; **plasma Aβ42 was sex adjusted; ***plasma NFL was age adjusted. In the MCI (Initial diagnosis): *LASSO regression retained: pTau217, GFAP, Aβ42/40 ratio, pTau181, plasma Aβ40, NFL and Age; **plasma Aβ42 and plasma Aβ42/40 ratio were sex adjusted.

#### 3.4.2 Subgroup Analysis and Model Calibration

In the MCI subgroup, pTau217 maintained high predictive performance (AUC 92.8%, sensitivity 91.5%, PPV 90%), suggesting robustness across diagnostic categories. Notably, a LASSO model incorporating age and multiple plasma biomarkers (pTau217, GFAP, pTau181, Aβ42/40, Aβ40, NFL) achieved an AUC of 98.9%, with 100% specificity and PPV—indicating no false positives. Model calibration plots (Figure S4A,B) showed strong agreement between predicted and observed probabilities, with low Brier scores (0.05), supporting model reliability. To enhance clinical relevance, we calculated PPV and NPV using both observed and literature‐based prevalence estimates (Figure S5A–F), highlighting how predictive values shift across diagnostic settings.

#### 3.4.3 Two‐Threshold Versus Single‐Threshold Approaches

**FIGURE 4:**
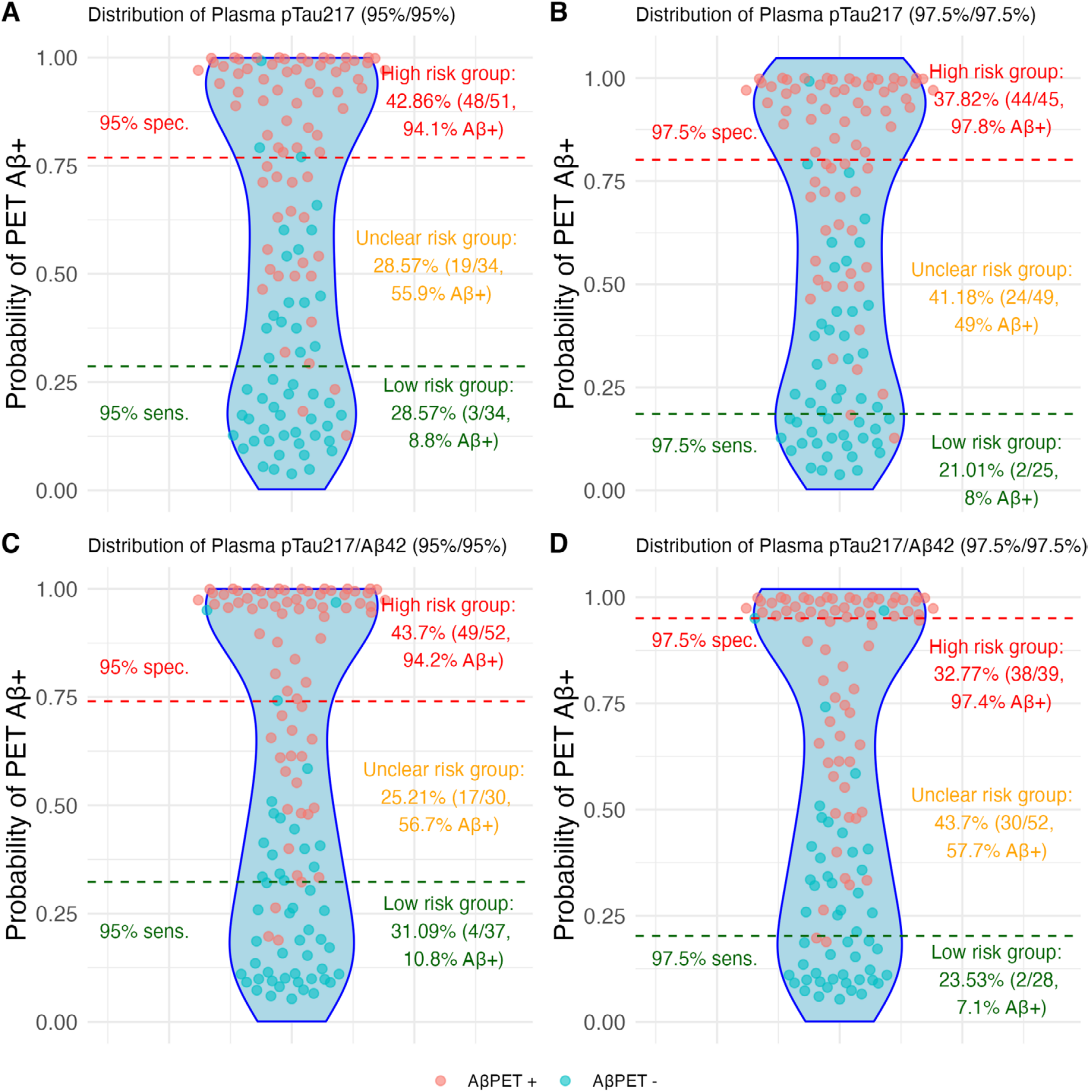
Two‐threshold cutoffs for predicting Aβ positivity in the brain in the whole clinical cohort (*n* = 122). Panels A and B show plasma pTau217 using lenient thresholds (95% Sensitivity and 95% specificity) and more conservative thresholds (97.5% Sensitivity and 97.5% specificity), respectively. 29% and 40% of subjects fall in the unclear risk group respectively. Panels C and D show plasma pTau217/Aβ42 using lenient thresholds (95% Sensitivity and 95% specificity) and more conservative thresholds (97.5% Sensitivity and 97.5% specificity), respectively. 25% and 44% of subjects fall in the unclear risk group respectively. Red dots depict Aβ+ subjects and blue dots Aβ− subjects according to visual reads.

**FIGURE 5:**
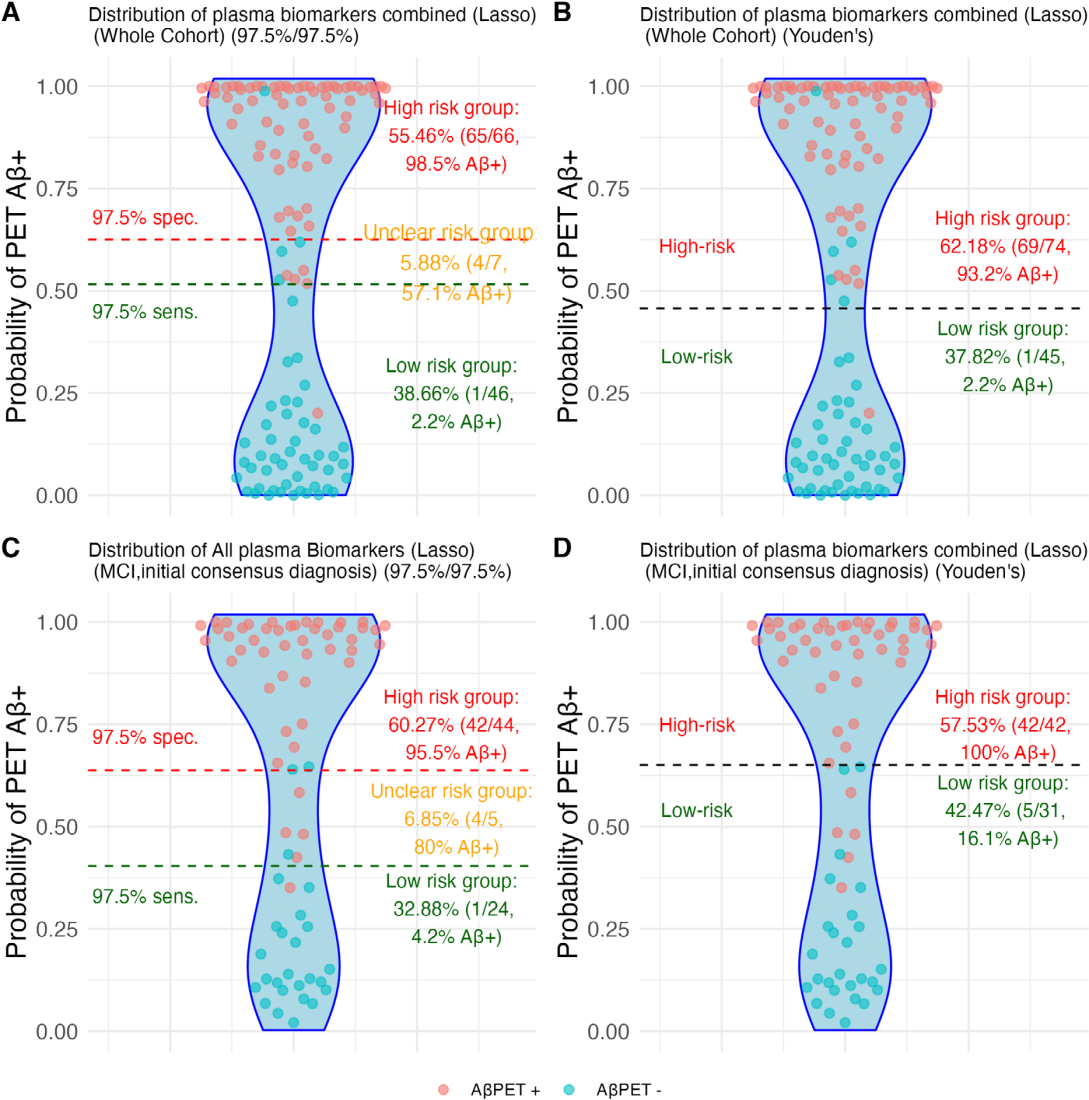
Comparison of two‐threshold and single‐threshold approaches for predicting Aβ positivity in the brain using a LASSO‐based combination of plasma biomarkers. Panels A and B display results from the full cohort (*n* = 122). Using conservative thresholds (97.5% sensitivity and specificity), only 6% of participants fell into the uncertain risk zone (Panel A). With a single‐threshold approach (Panel B), only 3 false positives were observed in the high‐risk group. Panels C and D show results for the MCI subgroup based on initial diagnosis (*n* = 73). Under the two‐threshold model (Panel C), 7% of individuals were classified as uncertain. Notably, the single‐threshold model (Panel D) achieved perfect classification in the high‐risk group, with 0 false positives. Red dots represent Aβ+ individuals and blue dots represent Aβ– individuals, as determined by visual PET reads.

Figure 4 illustrates the performance of plasma pTau217 and pTau217/Aβ42 ratio in predicting Aβ pathology using a two‐threshold approach, with both conservative (97.5%) and lenient (95%) sensitivity/specificity cutoffs. The lenient thresholds resulted in uncertain classification zones for 29% (pTau217) and 25% (pTau217/Aβ42), respectively. In these high‐risk groups, both biomarkers produced only 3 false positives—an improvement over the single‐threshold method, which yielded 8 and 5 false positives, respectively. Figure 5 extends this comparison to the LASSO‐based biomarker combination, showing excellent performance with just 1 false positive using the two‐threshold method. Interestingly, in the MCI subgroup, the single‐threshold approach outperformed the two‐threshold method, achieving 0 false positives versus 2.

#### 3.4.4 Zone Classification and Clinical Workflow

To address diagnostic uncertainty in the gray zone identified by the two‐threshold pTau217 model (*n* = 49), we applied a LASSO‐based multivariable classifier. This two‐step strategy—using simple thresholds for clear cases and a more complex model for ambiguous ones—mirrors a realistic clinical workflow. The LASSO model performed exceptionally well in this subgroup, achieving an AUC of 0.995, sensitivity of 1.00, and specificity of 0.92. It correctly classified 24 true positives and 23 true negatives, with only 2 false positives and no false negatives. These results suggest that targeted, data‐driven modeling can significantly enhance diagnostic precision in cases where single‐biomarker thresholds are inconclusive.

**FIGURE 6:**
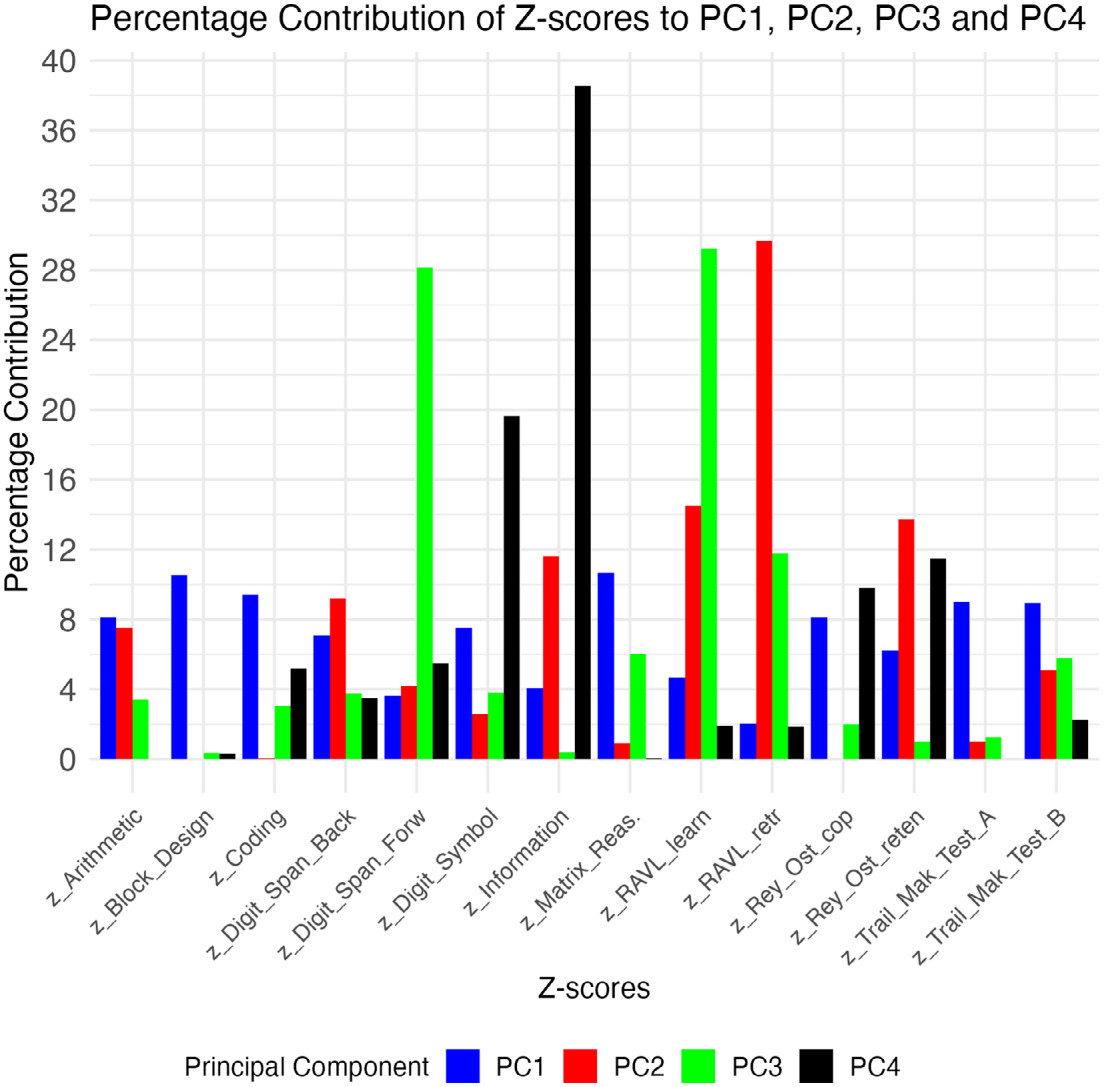
Percentage contribution of all 14 NP tests to PCs, scaled to the total variance. PC1‐4 have been labeled respectively as: “global indicator of disease,” “memory encoding and recall,” “verbal memory,” and “knowledge and semantic memory.”

### 3.5 Principal Component Analysis

*InDaPCA* was ran on the 14 neuropsychological tests available in 88 subjects, who are reported in Supplementary Table S1, and 4 principal components (PC) were retained according to Kaiser Criterion. Figure 6 shows the percentual contribution of each neuropsychological test to the total variance of each of the four components retained.

PC1 has been labelled as “global indicator of disease” since there was not a clear predominance of contribution among the tests considered. PC2 has been labelled as “memory encoding and recall” given the high contribution of RAVL retrieval test (30%) to this component. PC3 has been labelled “verbal memory” given the relatively higher contributions of Digit Span Forward (28%) and RAVL learning (29%) tests and PC4 has been labelled “knowledge and semantic memory” given the higher contribution of Information (39%) and Digit symbol (20%) tests.

#### 3.5.1 PCA Component Trends Across Diagnostic Groups

**FIGURE 7:**
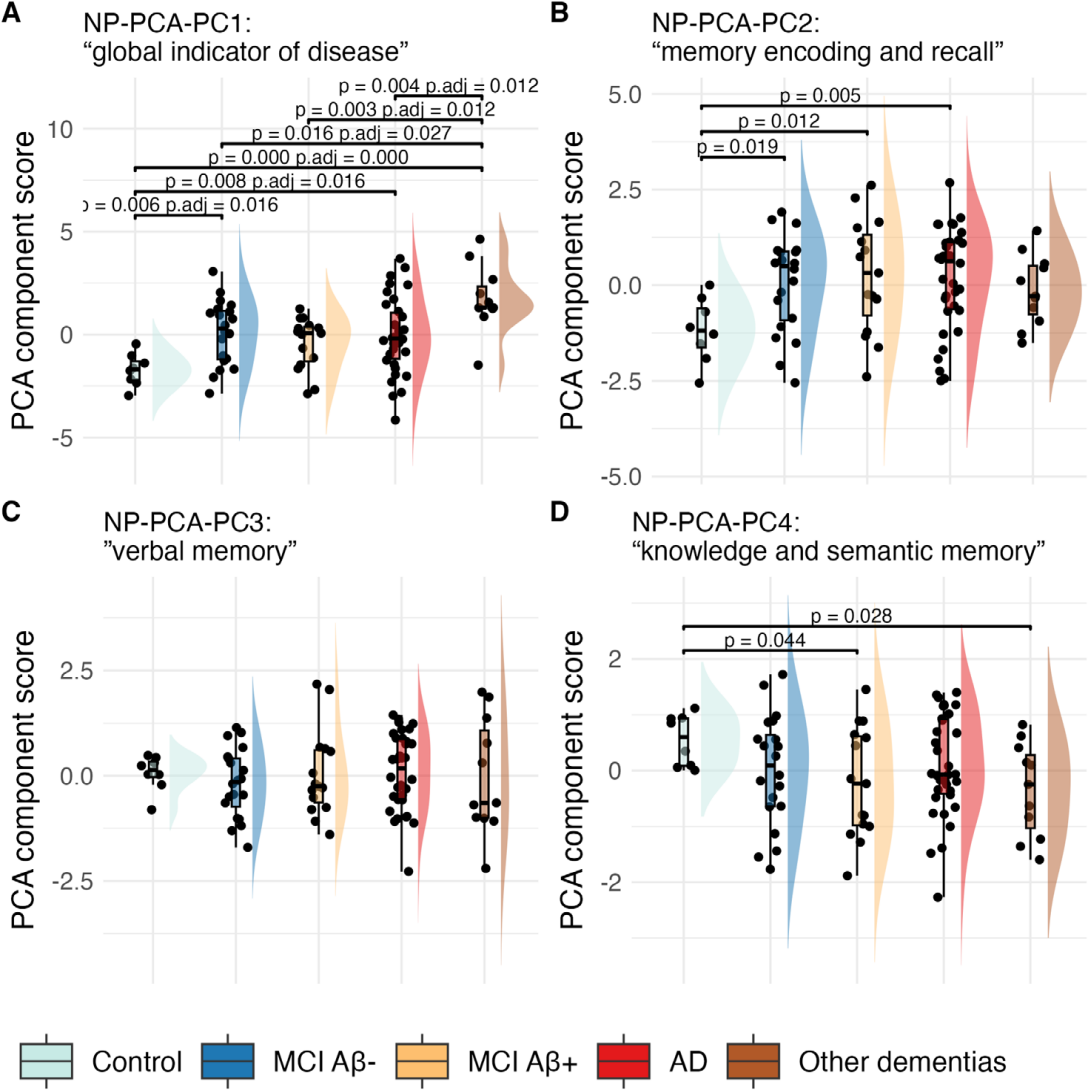
Principal components (PCs) from principal component analysis (PCA) of NP tests across diagnostic groups. PC1 and PC2 show a progression along with an increase in disease severity.

Figure 7 shows PCA component scores by diagnostic groups. PC1 and PC2 scores increased progressively with disease severity, suggesting they capture dimensions related to neurodegeneration and cognitive decline. In contrast, PC3 showed no significant variation across groups, while PC4 exhibited a non‐significant trend toward lower scores with increasing disease severity. Among these, only PC1—associated with global disease status and fluid intelligence—showed statistically significant group differences after FDR correction. Additionally, PC1 (adjusted for education) correlated negatively with MMSE (also adjusted) (*ρ* = −0.3 and *p* = 0.0072), supporting its relevance to cognitive impairment. In the MCI Aβ+ subgroup, PC2 was positively correlated with brain Aβ levels (Centiloid; *ρ* = 0.68, *p* = 0.0069), indicating a potential link between this component and amyloid pathology.

#### 3.5.2 Key Contributors to PCA Components

Figure S1 highlights major contributors to each principal component by diagnostic group. Matrix Reasoning (PC1) and Rey‐Osterrieth Retention (PC2) showed the lowest *z*‐scores in the “Other dementias” group. However, Rey‐Osterrieth Retention scores were not significantly different between the “Other dementias” and AD groups, though both were lower than controls. For PC3 and PC4, RAVL retrieval and Digit Symbol scores were highest in the control group, suggesting these components may reflect preserved memory and processing speed, respectively.

### 3.6 Correlations Between Plasma Biomarkers With PCA Components and Major NP Test Contributors (Post Amy‐PET Diagnosis)

#### 3.6.1 Correlations Between Plasma Biomarkers and PCA Components

**FIGURE 8:**
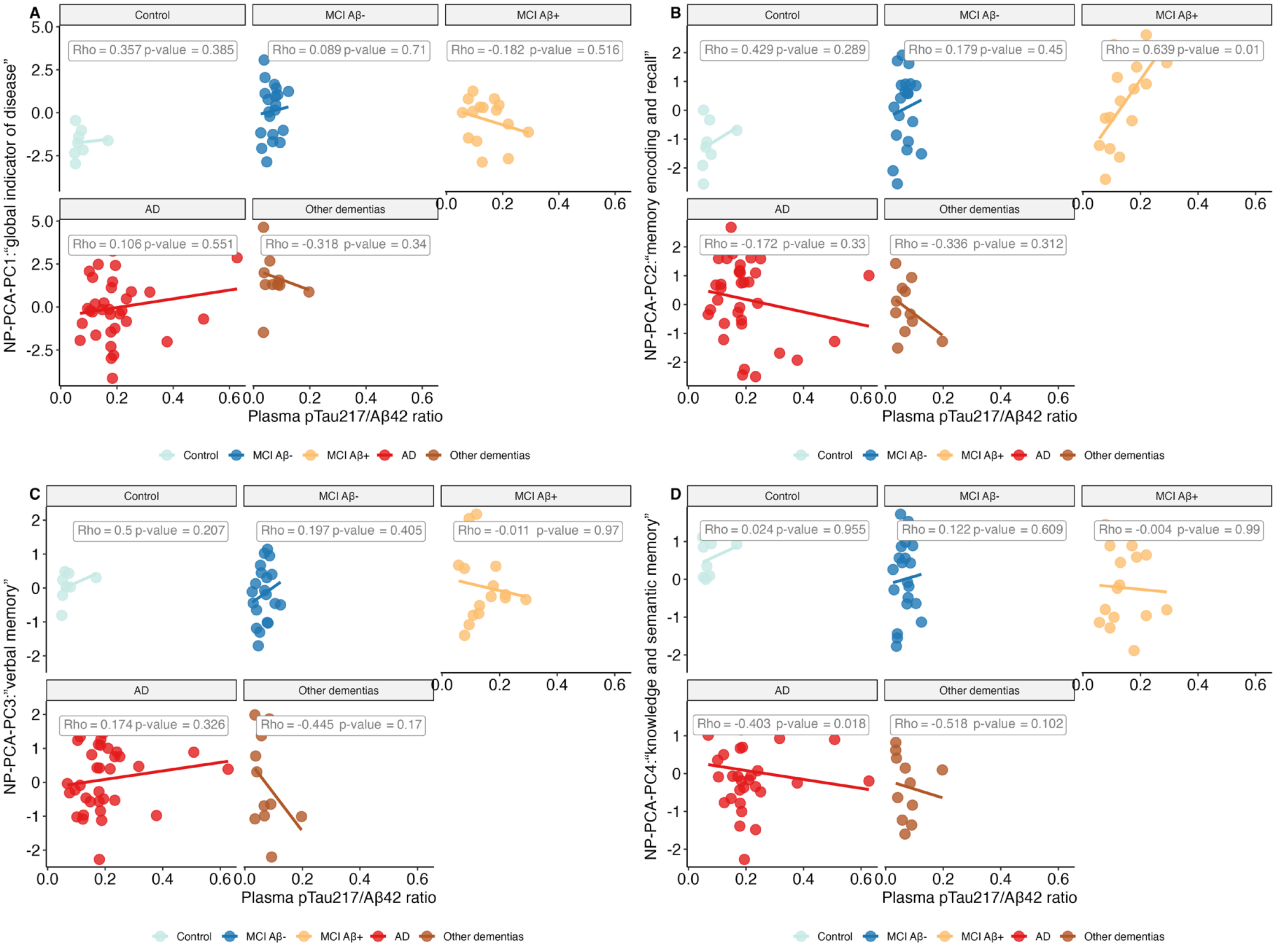
Associations between plasma pTau217/Aβ42 ratio and Principal Components (PCs) from PCA across diagnostic groups (Post Amy‐PET diagnosis). In the MCI Aβ+ group, plasma pTau217/Aβ42 ratio was significantly associated with PC2: “memory encoding and recall.” A secondary association was observed between the ratio and PC4 (knowledge and semantic memory) in the AD group, though this may be influenced by outliers. All associations shown are unadjusted for covariates.

Figure 8 and Figure S2 present correlations between unadjusted PCA component scores and plasma biomarkers (pTau217/Aβ42 ratio and pTau217) across diagnostic groups (control, MCI Aβ−, MCI Aβ+, AD and “Other dementias” based on Post Amy‐PET diagnosis). Plasma pTau217 did not show statistically significant correlations with any PCA components in any group (Figure S2), although a non‐significant trend was observed between plasma pTau217 and PC2 in the MCI Aβ+ group. In contrast, the pTau217/Aβ42 ratio showed a significant positive correlation with PC2 in the same group (Figure 8), suggesting a stronger association with cognitive dimensions captured by this component.

#### 3.6.2 Correlations Between Plasma Biomarkers and Key Individual Tests

**FIGURE 9:**
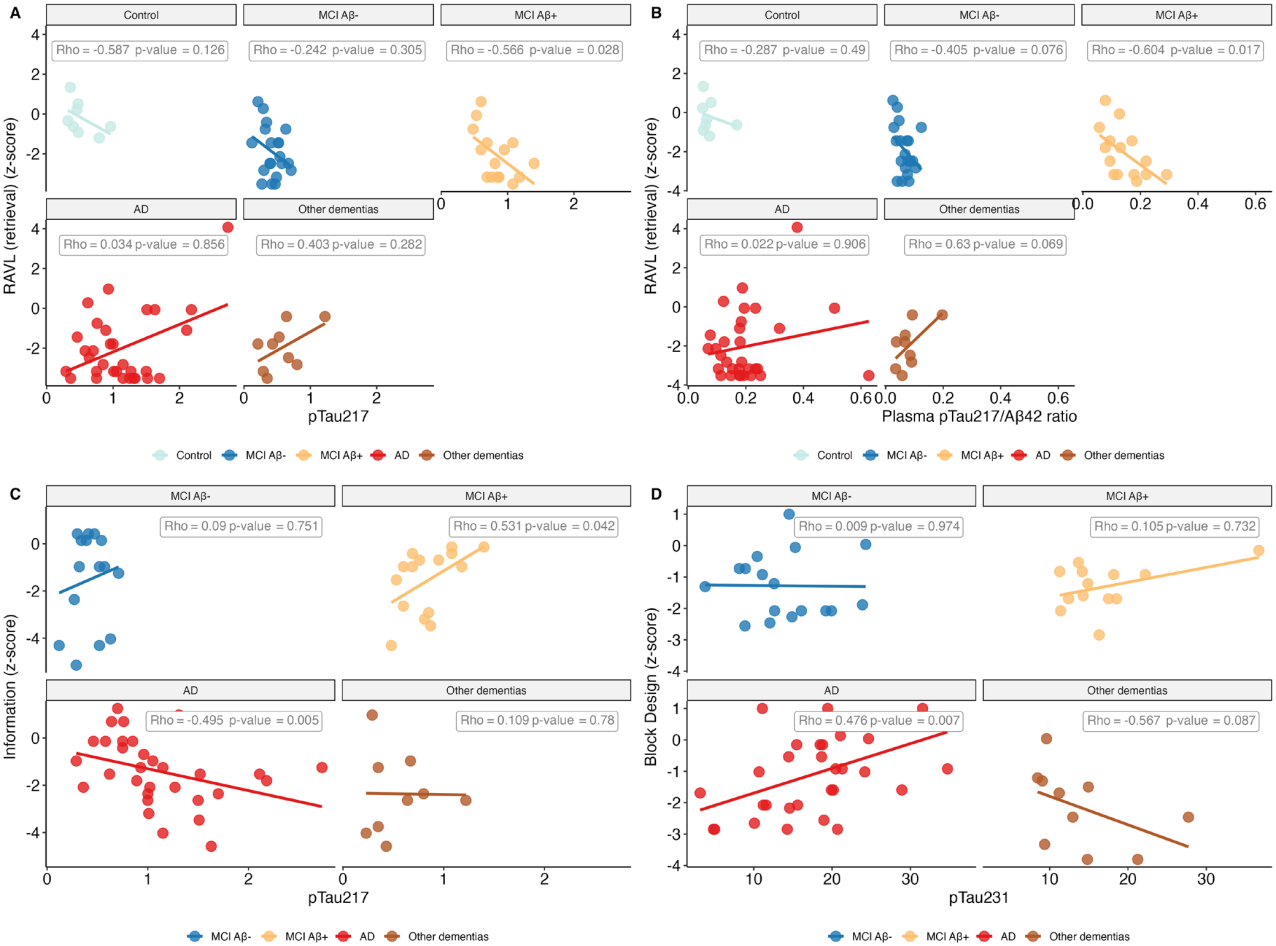
Associations between plasma biomarkers and major contributors to Principal Components (PCs) across diagnostic groups (Post Amy‐PET diagnosis). RAVL retrieval (*z*‐score) is negatively associated with plasma pTau217 (panel A) and the pTau217/Aβ42 ratio (panel B) in the MCI Aβ+ group. Plasma pTau217 is also positively and negatively related to Information (*z*‐score) in the MCI Aβ+ and AD groups, respectively (panel C). Block design (*z*‐score) was positively related to plasma pTau231 in the AD group (panel D). The associations shown in the plots are unadjusted for covariates.

Figure 9 highlights further associations between plasma biomarkers and cognitive domains. Plasma pTau217 was associated with the Information subtest (PC4) in both the MCI Aβ+ and AD groups; however, these associations were mediated by age. Additionally, plasma pTau231 was significantly associated with Block Design (PC1) in the AD group, indicating a potential link between the biomarker and visuospatial reasoning in advanced disease stages.

Table 3 reports significant associations (*p* < 0.01) between plasma biomarkers and individual cognitive tests that contributed most to the PCA components. Notably, in the control group, plasma NFL showed a strong negative correlation with RAVL learn (education‐corrected) with *ρ* = −0.88 and *p* = 0.0072 (Figure S3). These associations were adjusted (when necessary) for relevant confounders, ensuring that observed relationships reflect true biological links rather than demographic effects. Among the biomarkers examined, plasma pTau217 consistently emerged as a key correlate of cognitive performance, particularly in early disease stages.

**TABLE 3:** Correlations between plasma biomarkers and individual NP tests by groups.

Group	Plasma biomarker	Cognitive test	*ρ*	*p*
Control	NFL	RAVL learning (educ. corrected)	−0.88	0.0072
MCI Aβ−	NFL	Information (age, sex corrected)	0.67	0.0064
MCI Aβ−	Aβ42	Matrix reasoning (uncorrected)	0.74	0.0039
MCI Aβ+	Aβ42	Digit span forward (sex corr.)	−0.73	0.0046
AD	NFL	RAVL learning (uncorrected)	−0.53	0.0024

#### 3.6.3 Biomarkers‐Cognition Relationships Across Diagnostic Groups

Figure 9 illustrates relationships between plasma biomarkers (pTau217, pTau217/Aβ42 ratio and pTau231) and some of the major contributors to PCA Components across diagnostic groups (based on Post Amy‐PET diagnosis). In the MCI Aβ+ group, lower RAVL Retrieval scores (*z*‐score; major contributor to PC2) were significantly associated with higher levels of plasma pTau217 and pTau217/Aβ42 ratio. Additionally, plasma pTau217 showed a differential relationship with Information scores (*z*‐score), being positively associated in MCI Aβ+ and negatively in the AD group, respectively (Figure 9C). Plasma pTau231 was positively associated with Block Design scores (*z*‐score; major contributor to PC1) in the AD group, suggesting a link between this biomarker and visuospatial reasoning. These findings reinforce the role of plasma pTau217 as a sensitive marker of cognitive decline, particularly in relation to memory and general knowledge domains.

## 4 Discussion

The pursuit of non‐invasive diagnostic tools for Alzheimer's disease (AD) has increasingly focused on plasma biomarkers capable of predicting brain amyloid (Aβ) load and reflecting cognitive impairment. Our study contributes to this effort by evaluating the diagnostic utility of plasma phosphorylated tau (pTau217) and its ratio with Aβ42 in a memory clinic setting.

In recent years, plasma pTau217 has emerged as a promising biomarker for AD, with studies such as those by Ashton et al. (2024) and Lehmann et al. (2024) highlighting its diagnostic accuracy. Our study builds on this foundation by demonstrating that plasma pTau217 can effectively distinguish MCI Aβ‐negative from AD, and its ratio with Aβ42 also differentiates MCI Aβ‐positive from AD. This finding is significant as it provides a practical tool for clinicians to identify different stages of cognitive impairment, enhancing the precision of diagnoses in memory clinics.

To assess predictive performance, we conducted ROC‐AUC analysis, showing that plasma pTau217 and its ratio with Aβ42 predict amyloid load positivity with high accuracy. Aβ load in the brain predicted by plasma biomarkers has been previously investigated by our (Bucci et al. 2023) and other groups (Mattsson‐Carlgren et al. 2024; Ashton et al. 2024, 2021; Mendes et al. 2024). Compared to our previous investigation, we have included plasma pTau217 and pTau217/Aβ42 ratio and implemented an improved statistical method to model the data (nested LOOCV). Our results show that plasma pTau217 and pTau217/Aβ42 ratio are superior to other plasma biomarkers and that the combination with other biomarkers can achieve PPV and Specificity of 100% in the MCI subgroup. Importantly, we found that a single‐threshold approach reduced the number of individuals in the diagnostic “gray zone,” addressing a key limitation of previous studies that relied on dual‐threshold models. Previous studies have recently relied on dual thresholds, which can lead to ambiguity in risk classification. Our findings suggest that a single threshold approach is not only practical but an acceptable trade‐off that enhances diagnostic clarity, making it a valuable tool for clinicians. In summary, our findings support plasma pTau217 and its Aβ42 ratio as highly accurate, non‐invasive biomarkers for detecting brain amyloid pathology. Furthermore, our study clearly demonstrates that plasma pTau217 can significantly distinguish mild cognitive impairment (MCI) Aβ‐negative from AD, and pTau217/Aβ42 clearly importantly differentiates MCI Aβ‐positive (prodromal AD) from AD dementia.

When considering the important point regarding the dependence of PPV and NPV on disease prevalence, we followed a published approach that adjusts predictive values based on setting‐specific prevalence estimates. By incorporating both literature‐based and cohort‐specific prevalence rates, we provide a more nuanced understanding of test performance across different clinical contexts. These analyses included in the Supporting Information highlight the potential utility of plasma biomarkers in screening and diagnostic workflows and how PPV is high mostly when the prevalence of amyloid positivity is high (as expected) and vice versa for NPV. These results also emphasize the need for careful interpretation of predictive values in real‐world settings and being considerate of the prevalence of the target population.

While the high AUC values obtained with LASSO regression aimed at combining different plasma biomarkers are promising, we recognize the potential risk of overfitting, particularly given the modest sample size and lack of external validation. To address this, we conducted additional analyses to evaluate model calibration and overall predictive accuracy. The calibration plots (Figure S4A,B) demonstrate strong agreement between predicted and observed probabilities, and the Brier scores indicate more than acceptable predictive performance. These findings provide further support for the robustness of the models beyond discrimination alone. However, we emphasize that these results should be interpreted with caution and validated in independent cohorts to confirm generalizability. While combining biomarkers improves prediction accuracy, our study highlights the trade‐off between diagnostic precision and practicality. This nuanced understanding is crucial for clinical decision‐making, as it emphasizes the need for cost‐effective and accessible diagnostic tools (Eisenberg 1995). Eisenberg (1995) stressed the importance of balancing these factors, and our findings provide empirical support for this perspective.

To bridge the gap between diagnostic precision and cognitive assessment, we applied Principal Component Analysis (PCA) to 14 neuropsychological tests. This data‐driven approach identified four principal components (PCs), with PC1 and PC2 showing a progressive increase across the Alzheimer's disease (AD) continuum, indicating their relevance to disease severity. Notably, PC2—interpreted as reflecting memory encoding and recall—was significantly associated with the plasma pTau217/Aβ42 ratio in the MCI Aβ+ group, consistent with recent findings (Lehmann et al. 2025). These associations, particularly with cognitive tests such as RAVL retrieval and Information, offer insight into the specific domains most affected by AD pathology (Arranz et al. 2024).

Our findings align with those of Fernández Arias et al. (2025), who reported strong associations between plasma pTau217 and memory composite scores, even after adjusting for multiple covariates. Notably, the authors corrected both terms of the correlations for all available covariates (age, sex, APOE ε4, years of education, and Fazekas scores to exclude vascular neurodegenerative disease) (Fernández Arias et al. 2025). In contrast, we adopted a conservative approach, avoiding overcorrections (Pourhoseingholi et al. 2012). Nonetheless, we confirm that memory related test scores such as low RAVL‐retrieval scores are associated with high levels of plasma pTau217 and we extend these findings to the pTau217/Aβ42 ratio. This is in line with a recent study from Vanderlip and Stark (2025). Furthermore with our data‐driven approach, we observed that the plasma pTau217/Aβ42 ratio was also directly associated with the PCA‐PC2: “memory encoding and recall” scores in the MCI Aβ+ group. The direction of the relationship—where higher ratios correspond to higher component scores—does not necessarily imply an improvement in cognition. As illustrated in Figure 7, both PC1 and PC2 scores progressively increase when moving from a group with no pathology to one with more severe pathology, as supported by a longitudinal study (Chang et al. 2022). This indicates that the variability captured by PCA translates into positive scores when cognitive performance is impaired. Supporting this interpretation, PC1 is negatively related to MMSE scores across the entire group. Together, these results reinforce the link between plasma pTau217‐related biomarkers and memory impairment, and demonstrate that PCA can effectively capture cognitive dimensions aligned with AD pathology.

If via RAVL‐retrieval we assess verbal memory and retrieval, and with the Information subtest we assess semantic memory, then the observed pattern—where lower Information *z*‐scores are associated with higher plasma pTau217 levels in AD, but with lower pTau217 in the MCI Aβ+ group—can be interpreted by considering the cognitive reserve framework (Vockert et al. 2024). Although we do not have a direct measure of cognitive reserve, the Information *z*‐score has been linked to both cognitive performance and reserve capacity (Corujo‐Bolaños et al. 2023). It is plausible that individuals in the MCI Aβ+ group, who are still in the earlier stages of disease progression, are able to recruit compensatory mechanisms—supported by their educational or cognitive background—to maintain semantic memory performance despite moderate pathology. In contrast, AD subjects may have reached a threshold where compensatory mechanisms are no longer sufficient, leading to more pronounced cognitive decline and a stronger association between semantic memory impairment and pTau217 levels.

This interpretation aligns with findings from population‐based studies in Sweden, where higher education—used as a proxy for cognitive reserve—has been associated with delayed onset of dementia symptoms despite similar levels of neuropathology (e.g., IGEMS [Interplay of Genes and Environment across Multiple Studies] Consortium [Pedersen et al. 2019]). In our cohort, educational level ranged widely from 5 to 21 years, reflecting the diversity of individuals referred to our memory clinic. This variation includes patients with limited formal education as well as those requiring translation support, underscoring the real‐world complexity of clinical populations. Importantly, this heterogeneity was not controlled for in recruitment but rather reflects the naturalistic setting of the study and the population we serve.

The broader range of cognitive impairment observed in AD (reflected by more negative Information *z*‐scores) and the corresponding elevation in pTau217 levels may reflect the breakdown of these compensatory processes. Furthermore, our findings support the notion that changes in semantic memory can be detected via plasma pTau217, in line with Arias and colleagues' work (Fernández Arias et al. 2025), and contribute to a more nuanced understanding of how biomarker levels relate to cognitive function across disease stages.

Our study has several limitations, due to its clinical nature and the real‐world setting in which data were acquired. Specifically, we had a small sample size and incomplete data, which necessitated the implementation of the *InDaPCA* method for handling missing values. We have applied rigorous approaches to correct for confounders, although only age, sex, and education were available. Additionally, while the numerous correlations performed might have warranted a more stringent significance threshold, intending this as an exploratory study, we settled on *p* < 0.01 for the correlation tests. We acknowledge that the cognitive healthy control group is small, which reflects the clinical nature of this real‐world study, where the primary focus is on patients presenting with cognitive symptoms. The inclusion of an external subset from a research cohort, although limited, offers a valuable point of reference and helps contextualize the findings in the clinical groups. However, comparisons involving this control group should be interpreted with caution due to differences in recruitment setting and sample size.

## 5 Conclusions

Our study highlights the importance of plasma pTau217 and plasma pTau217/Aβ42 ratio as promising biomarkers for early diagnosis of AD and for predicting amyloid plaque pathology in the brain. These biomarkers were associated with cognitive test outcomes and data‐driven measures in a real‐world memory clinic cohort, which included a relatively young population—a group that remains underrepresented in biomarker clinical research. Importantly, our study integrates plasma biomarkers with amyloid PET, neuropsychological assessments, and CSF (used here for clinical diagnosis only) in a clinical setting, offering a comprehensive view that is rarely achieved outside of the research‐focused cohorts. While these findings support the potential clinical utility of plasma pTau217 and its ratio with Aβ42, we acknowledge that further validation is needed before routine implementation. Specifically, standardized thresholds, external replication, and practical models for clinical integration must be established.

The use of blood‐based biomarkers can be considered a promising avenue due to their accessibility, lower cost, and less invasive nature compared to traditional diagnostic methods. However, their adoption in clinical practice should be approached cautiously. Our results suggest that plasma pTau217 may be the most informative when interpreted alongside clinical information, particularly cognitive status (e.g., MCI vs. other groups), to enhance diagnostic accuracy. Future research should aim to replicate these findings in larger and more diverse populations and explore the added value of combining neuropsychological tests with advanced neuroimaging and multimodal biomarker approaches (Dubois et al. 2021). Longitudinal studies investigating the time course of amyloid deposition, plasma biomarkers, and cognitive decline will be essential for refining disease progression models and optimizing early intervention strategies—though we recognize the challenges of conducting such studies in the real‐world clinical setting.

## Author Contributions

**Marco Bucci:** conceptualization, methodology, software, data curation, writing – original draft, formal analysis, writing – review and editing, visualization. **Ove Almkvist:** conceptualization, methodology, investigation, data curation, writing – original draft, writing – review and editing. **Marina Bluma:** methodology, data curation, writing – review and editing. **Nicholas J. Ashton:** methodology, writing – review and editing. **Irina Savitcheva:** methodology, investigation, data curation, writing – review and editing. **Konstantinos Chiotis:** writing – review and editing. **Guglielmo Di Molfetta:** methodology, writing – review and editing. **Kaj Blennow:** methodology, writing – review and editing. **Henrik Zetterberg:** methodology, writing – review and editing. **Agneta Nordberg:** conceptualization, investigation, data curation, project administration, funding acquisition, supervision, writing – original draft, resources, writing – review and editing.

## Conflicts of Interest

Henrik Zetterberg has served on scientific advisory boards and/or as a consultant for Abbvie, Acumen, Alector, Alzinova, ALZpath, Amylyx, Annexon, Apellis, Artery Therapeutics, AZTherapies, Cognito Therapeutics, CogRx, Denali, Eisai, Enigma, LabCorp, Merry Life, Nervgen, Novo Nordisk, Optoceutics, Passage Bio, Pinteon Therapeutics, Prothena, Quanterix, Red Abbey Labs, reMYND, Roche, Samumed, Siemens Healthineers, Triplet Therapeutics, and Wave, has given lectures sponsored by Alzecure, BioArctic, Biogen, Cellectricon, Fujirebio, Lilly, Novo Nordisk, Roche, and WebMD, and is a co‐founder of Brain Biomarker Solutions in Gothenburg AB (BBS), which is a part of the GU Ventures Incubator Program (outside submitted work). Agneta Nordberg has served as a consultant for AG Lundbeck AB, Hoffman La Roche, and AVVA Pharmaceuticals, given lectures for Hoffman La Roche and Astra Zeneca, and served on the advisory board for Dementia Platform UK. The remaining authors declare no conflicts of interest.

## Peer Review

The peer review history for this article is available at https://www.webofscience.com/api/gateway/wos/peer‐review/10.1111/jnc.70182.

## Supporting information


**Data S1:** jnc70182‐sup‐0001‐supinfo.pdf.

## Data Availability

The data from this study can be obtained from the corresponding author upon reasonable request, and this is subject to standard data‐sharing agreements.
