# Supplementary material for: Profiling Plasma Biomarkers, Particularly pTau217 and pTau217/Aβ42, and Their Relation to Cognition in Memory Clinic Patients
Source: J Neurochem. 2025 Aug 11;169(8):e70182. doi: 10.1111/jnc.70182 (PMC12336780; doi:10.1111/jnc.70182)
Supplement: Supplementary file 1 — Data S1: jnc70182‐sup‐0001‐supinfo.pdf. [file JNC-169-0-s001.pdf]

TITLE:

Profiling plasma biomarkers, particularly pTau217 and pTau217/Aβ42, and their relation to cognition in memory clinic patients.

AUTHORS:

Marco Bucci, Ove Almkvist, Marina Bluma, Nicholas J. Ashton, Irina Savitcheva, Konstantinos Chiotis, Guglielmo Di Molfetta, Kaj Blennow, Henrik Zetterberg and Agneta Nordberg

Supplementary Material and Methods

Description of the box plots

These are the specifics of the box plots presented in the figures.

- **Lower edge**: First quartile (**Q1**, 25th percentile)
- **Upper edge**: Third quartile (**Q3**, 75th percentile)
- The **height** of the box is the **interquartile range (IQR)** = Q3 - Q1
- **Line inside the box**:
    - This is the **median** (50th percentile) of the data
- **Whiskers**:
    - Extend from the box to the **smallest and largest values** within **1.5 × IQR** from Q1 and Q3
- **Points outside the whiskers**:
    - These are **outliers**

Any data point beyond 1.5 × IQR from the quartiles is plotted individually

Supplemental Method on PCA calculations

InDaPCA was performed inputting neuropsychological test results (n=14) with a variable degree of missingness (6%-58%). Parallel analysis with 1000 simulations identified 2 PC to retain but Kaiser Criterion (eigenvalue > 1 retained) identified 4 PCs to retain.

The percentual contribution to the total variance of each test in each component was calculated with the formulas:

- total_variance <- sum(eigenvalues)
- variance_explained <- (eigenvalues / total_variance) * 100
- loadings_squared <- pca_loadings^2
- pc1_contrib <- loadings_squared[, 1] / sum(loadings_squared[, 1]) * 100
- pc1_contrib_scaled <- pc1_contrib * variance_explained[1] / 100

To assess which adjustment for covariates to perform on the PCs for the correlation analyses, we have adopted a special approach because adjusting directly the PC could have not been optimal in case the PCA process might have masked the influence of a covariate. Firstly, the covariate effects have been tested for the whole group and for all the diagnostic groups

separately on the individual tests before PCA (Gao *et al.* 2025), the results have been tabulated if significant in a summary where information of PCA analysis was present, like the top 70% contributors to each PC and if they variable had a low, moderate or high level of missingness. Secondly, the selection of the variables to adjust was done according to this algorithm: 1) Covariate affects >=50% of the top contributing features (and most have <30% missingness), then adjust for such covariate; 2) Covariate affects the top 3 highest-loading features, including at least one low-missingness feature, then adjust; 3) Covariate affects only weak contributors, do not adjust; 4) Covariate is significant only in feature with > 30% missingness, do not adjust. Third step was to adjust. Fourth step, after checking the correlations PC unadjusted vs PC adjusted, if Rho was >0.95, the adjustment was deemed as unnecessary/ineffective and discarded.

Supplementary Table 1 – General characteristics of the study groups where NP data was available

Variable	Control N = 8	MCI Aβ- N = 20	MCI Aβ+ N = 15	AD N = 34	Other dementias N = 11	All N=88	p-value*
Age	-	-	-	-	-		0.693¹
Mean (SD)	68 (8)	65 (9)	66 (7)	64 (7)	63 (5)	65 (8)	
Min, Max	58, 81	44, 83	56, 80	48, 83	57, 73	44, 83	
Sex	-	-	-	-	-		0.1²
F	4 (50%)	11 (55%)	13 (87%)	20 (59%)	4 (36%)	52 (59%)	
M	4 (50%)	9 (45%)	2 (13%)	14 (41%)	7 (64%)	36 (41%)	
Education	-	-	-	-	-		0.193¹
Mean (SD)	13.4 (3.7)	12.4 (2.3)	13.8 (3.9)	12.8 (3.1)	10.8 (2.4)	12.7 (3.1)	
Min, Max	11.0, 21.0	9.0, 17.0	7.0, 20.0	5.0, 17.0	7.0, 15.0	5.0, 21.0	
Missing	0	1	0	0	0	1	
Centiloid		cd	be	be	cd		<0.001¹
Mean (SD)		-1 (16)	77 (26)	89 (25)	6 (19)	54 (47)	
Min, Max		-23, 38	33, 121	38, 150	-16, 43	-23, 150	
Missing	8	2	0	1	0	11	
MMSE	-	-	-	-			0.454¹
Mean (SD)		26.4 (2.5)	26.9 (1.8)	26.3 (3.1)	25.1 (2.7)	26.3 (2.7)	
Min, Max	28.0, 30.0	21.0, 30.0	24.0, 30.0	17.0, 30.0	20.0, 29.0	17.0, 30.0	
Missing	8	0	0	1	1	10	
Amyloid Beta positivity (VR)		cd	be	be	cd		<0.001²
Aβ PET-		20 (100%)	1 (6.7%)	0 (0%)	10 (91%)	31 (39%)	
Aβ PET+		0 (0%)	14 (93%)	34 (100%)	1 (9.1%)	49 (61%)	
Missing	8	0	0	0	0	8	

* ¹ Kruskal-Wallis test, ² Chi-Square test. VR=Visual Read. CLD letters (a,b,c,d) indicate significant differences between groups. Letters b, c, d, e indicate respectively difference with MCI Aβ-, MCI Aβ+, AD, Other dementias group

Supplementary Table 2 – Biomarkers by diagnostic group

Variable	MCI Aβ-<br>N = 29	MCI Aβ+<br>N = 19	AD<br>N = 51	Other dementias<br>N = 23	All<br>N=122	p-value*
pTau217 (pg/mL)	bc	ad	ad	bc		<0.001
Mean (SD)	0.39 (0.19)	0.82 (0.26)	1.15 (0.51)	0.49 (0.31)	0.79 (0.50)	
Min, Max	0.08, 0.82	0.41, 1.40	0.29, 2.73	0.14, 1.39	0.08, 2.73	
Missing	0	0	1	1	2	
pTau217/Aβ42 (ratio)	bc	acd	abd	bc		<0.001
Mean (SD)	0.07 (0.03)	0.14 (0.06)	0.20 (0.10)	0.07 (0.05)	0.13 (0.10)	
Min, Max	0.01, 0.17	0.06, 0.29	0.07, 0.63	0.02, 0.20	0.01, 0.63	
Missing	0	0	1	1	2	
pTau231 (pg/mL)	c		a			0.006
Mean (SD)	14 (6)	16 (6)	19 (8)	15 (7)	16 (7)	
Min, Max	2, 28	8, 37	3, 42	5, 28	2, 42	
Missing	1	0	1	1	3	
pTau181 (pg/mL)	c	c	abd	c		0.002
Mean (SD)	11 (6)	11 (4)	17 (20)	14 (18)	14 (16)	
Min, Max	3, 30	5, 24	4, 151	4, 95	3, 151	
GFAP (pg/mL)	bc	a	ad	c		<0.001
Mean (SD)	98 (48)	152 (53)	175 (74)	126 (63)	144 (70)	
Min, Max	35, 264	62, 280	60, 392	43, 296	35, 392	
NfL (pg/mL)	-	-	-	-		0.4
Mean (SD)	23 (17)	19 (6)	23 (10)	25 (12)	23 (12)	
Min, Max	7, 90	9, 31	7, 50	12, 60	7, 90	
Aβ42 (pg/mL)			d	c		0.006
Mean (SD)	6.63 (2.30)	6.45 (1.46)	6.12 (1.99)	7.85 (1.90)	6.62 (2.06)	
Min, Max	0.46, 11.06	3.45, 8.61	0.57, 13.43	5.52, 13.69	0.46, 13.69	
Aβ42/40 (ratio)	-	-	-	-		0.055
Mean (SD)	0.059 (0.014)	0.057 (0.010)	0.059 (0.030)	0.062 (0.009)	0.059 (0.021)	
Min, Max	0.031, 0.082	0.031, 0.074	0.031, 0.253	0.043, 0.080	0.031, 0.253	

* Kruskal-Wallis test. CLD letters (a,b,c,d) indicate significant differences between groups. Letters a, b, c, d indicate respectively difference with MCI Aβ-, MCI Aβ+, AD, Other dementias group

Supplementary Table 3 – *Z*-scores by diagnostic group

Variable	Control N = 8	MCI Aβ- N = 20	MCI Aβ+ N = 15	AD N = 34	Other dementias N = 11	All N=88	p-value[*]
RAVLT learning (z-score)	-	-	-	-	-		0.2
Mean (SD)	-0.46 (0.94)	-1.18 (0.86)	-1.30 (1.12)	-1.14 (0.99)	-1.52 (0.80)	-1.15 (0.97)	
Min, Max	-1.57, 0.93	-2.68, 0.68	-3.73, 1.31	-2.47, 0.89	-2.68, -0.37	-3.73, 1.31	
Missing	0	0	0	3	2	5	
RAVLT retrieval (z-score)	bcde	a	a	a	a		0.017
Mean (SD)	-0.20 (0.84)	-1.91 (1.24)	-2.07 (1.26)	-2.02 (1.77)	-1.98 (1.12)	-1.82 (1.50)	
Min, Max	-1.20, 1.34	-3.52, 0.62	-3.52, 0.62	-3.52, 4.07	-3.52, -0.41	-3.52, 4.07	
Missing	0	0	0	3	2	5	
Coding (z-score)	bde	a		a	a		0.002
Mean (SD)	0.23 (0.76)	-1.11 (1.11)	-0.72 (0.76)	-0.82 (0.87)	-1.62 (0.91)	-0.85 (1.00)	
Min, Max	-0.75, 1.66	-2.66, 1.96	-2.02, 0.29	-2.72, 0.93	-2.47, 0.22	-2.72, 1.96	
Missing	0	4	2	6	2	14	
Symbol Search (z-score)	bcde	a	a	a	a		0.005
Mean (SD)	0.13 (0.76)	-1.39 (1.07)	-1.54 (1.69)	-1.32 (1.54)	-2.24 (1.23)	-1.33 (1.46)	
Min, Max	-1.31, 1.26	-2.98, 0.36	-5.64, 1.02	-5.87, 2.13	-5.20, -0.76	-5.87, 2.13	
Missing	0	2	2	5	2	11	
Digit Span Backw (z-score)	-	-	-	-			0.066
Mean (SD)		-0.80 (0.80)	-0.42 (1.30)	-0.88 (0.78)	-1.50 (0.64)	-0.87 (0.93)	
Min, Max		-1.66, 0.76	-1.66, 2.37	-2.47, 0.76	-2.47, -0.05	-2.47, 2.37	
Missing	8	4	2	6	1	21	
Digit Span Forw (z-score)	-	-	-	-			0.5
Mean (SD)		-0.69 (1.03)	-0.74 (0.87)	-0.98 (0.98)	-1.10 (0.76)	-0.88 (0.94)	
Min, Max		-2.29, 1.38	-2.29, 0.46	-2.29, 1.38	-2.29, 0.46	-2.29, 1.38	
Missing	8	4	2	6	1	21	
Arithmetic (z-score)	-	-	-	-			0.3
Mean (SD)		-1.60 (0.96)	-1.20 (1.10)	-1.33 (1.20)	-1.74 (0.87)	-1.43 (1.07)	
Min, Max		-2.97, 0.37	-2.30, 1.37	-2.97, 2.03	-2.63, 0.37	-2.97, 2.03	
Missing	8	9	5	11	2	35	
Matrix reasoning (z-score)			e	e	cd		0.005
Mean (SD)		-1.21 (1.05)	-0.37 (0.72)	-1.06 (1.49)	-3.00 (0.83)	-1.18 (1.37)	
Min, Max		-2.63, 0.05	-1.66, 0.54	-3.12, 1.02	-4.10, -1.90	-4.10, 1.02	
Missing	8	7	4	11	5	35	
Block Design (z-score)	-	-	-	-			0.2
Mean (SD)		-1.28 (1.03)	-1.31 (0.72)	-1.08 (1.26)	-2.15 (1.25)	-1.32 (1.16)	
Min, Max		-2.56, 1.00	-2.85, -0.15	-2.85, 1.00	-3.81, 0.04	-3.81, 1.00	
Missing	8	3	2	3	1	17	
Information (z-score)	-	-	-	-			0.4
Mean (SD)		-1.51 (2.00)	-1.56 (1.36)	-1.40 (1.43)	-2.36 (1.73)	-1.58 (1.59)	
Min, Max		-5.14, 0.42	-4.31, -0.14	-4.58, 1.25	-4.58, 0.97	-5.14, 1.25	
Missing	8	5	0	3	2	18	
ROCFT copying (z-score)	e	e	e	e	abcd		0.005
Mean (SD)	-0.60 (0.89)	-1.85 (1.85)	-1.44 (1.73)	-1.37 (1.75)	-3.79 (2.06)	-1.71 (1.91)	
Min, Max	-1.93, 0.48	-7.34, 0.17	-5.42, 0.52	-6.29, 0.52	-8.04, -1.22	-8.04, 0.52	
Missing	0	2	1	4	1	8	
ROCFT retention (z-score)	de			a	a		0.011
Mean (SD)	-0.46 (0.72)	-1.12 (1.16)	-0.96 (1.35)	-1.51 (1.00)	-2.04 (0.89)	-1.29 (1.13)	
Min, Max	-1.78, 0.35	-2.89, 1.41	-2.72, 2.10	-2.89, 1.24	-2.89, -0.31	-2.89, 2.10	
Missing	0	2	3	5	1	11	
Trail Making Test A (z-score)	bd	a		a			0.013
Mean (SD)	0.77 (0.43)	-2.19 (2.59)	-0.19 (1.40)	-2.36 (4.64)	-3.31 (5.89)	-1.46 (3.67)	
Min, Max	0.02, 1.22	-5.57, 0.91	-2.52, 0.91	-16.96, 1.00	-13.54, 0.91	-16.96, 1.22	
Missing	0	12	8	21	6	47	
Trail Making Test B (z-score)	bcde	a	a	a	a		0.006
Mean (SD)	0.43 (0.86)	-2.87 (2.44)	-2.18 (1.71)	-3.13 (3.03)	-3.79 (4.05)	-2.29 (2.71)	
Min, Max	-1.08, 1.50	-7.46, -0.31	-4.76, -0.28	-10.62, -0.37	-7.77, 0.32	-10.62, 1.50	
Missing	1	12	7	21	8	49	

[*] Kruskal-Wallis test. RAVLT=Rey Auditory Verbal Learning Test, ROCFT=Rey-Osterrieth Complex Figure Test. CLD letters (a,b,c,d,e) indicate significant differences between groups. Letters a, b, c, d, e indicate respectively difference with Control, MCI Aβ-, MCI Aβ+, AD, Other dementias group

Supplementary Figure 1 – Some of the major contributors to Principal Components (PCs) from PCA by diagnostic groups

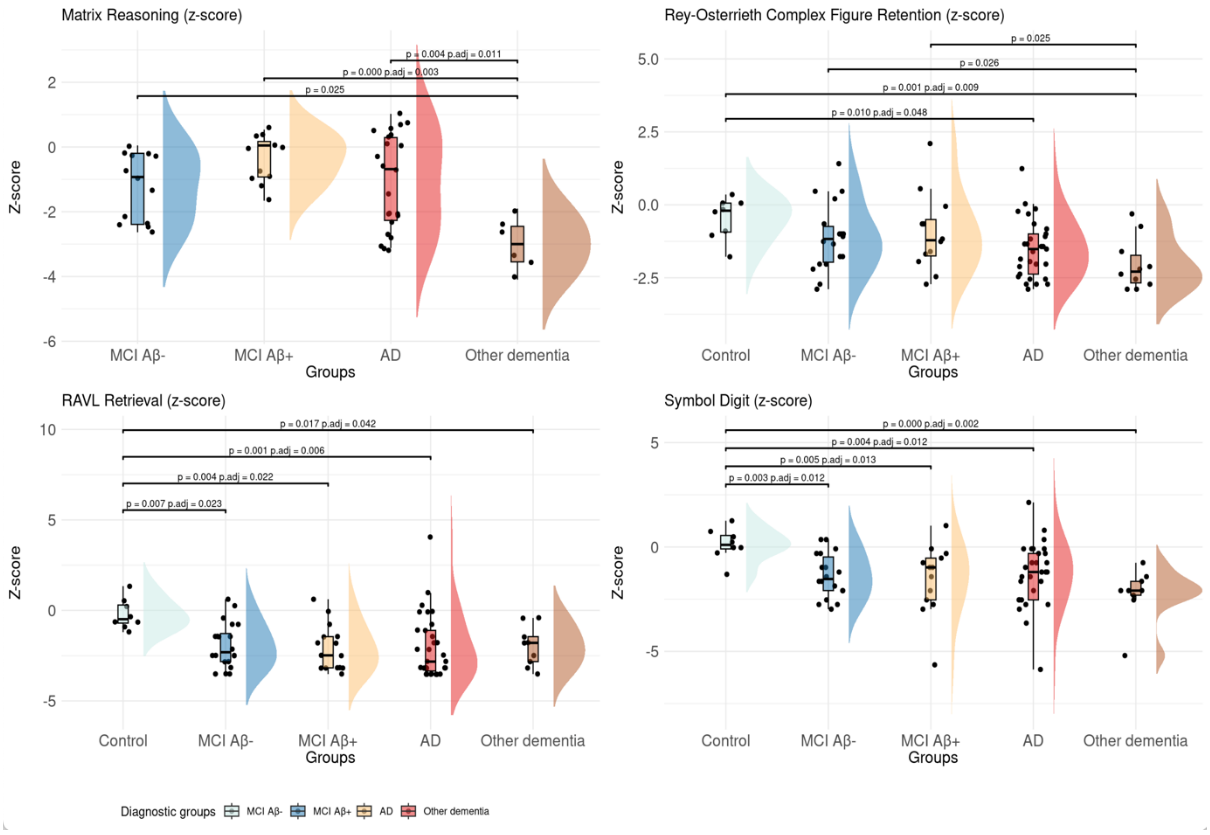

Supplementary Figure 2 – Principal Components (PCs) derived from PCA vs pTau217 by diagnostic groups

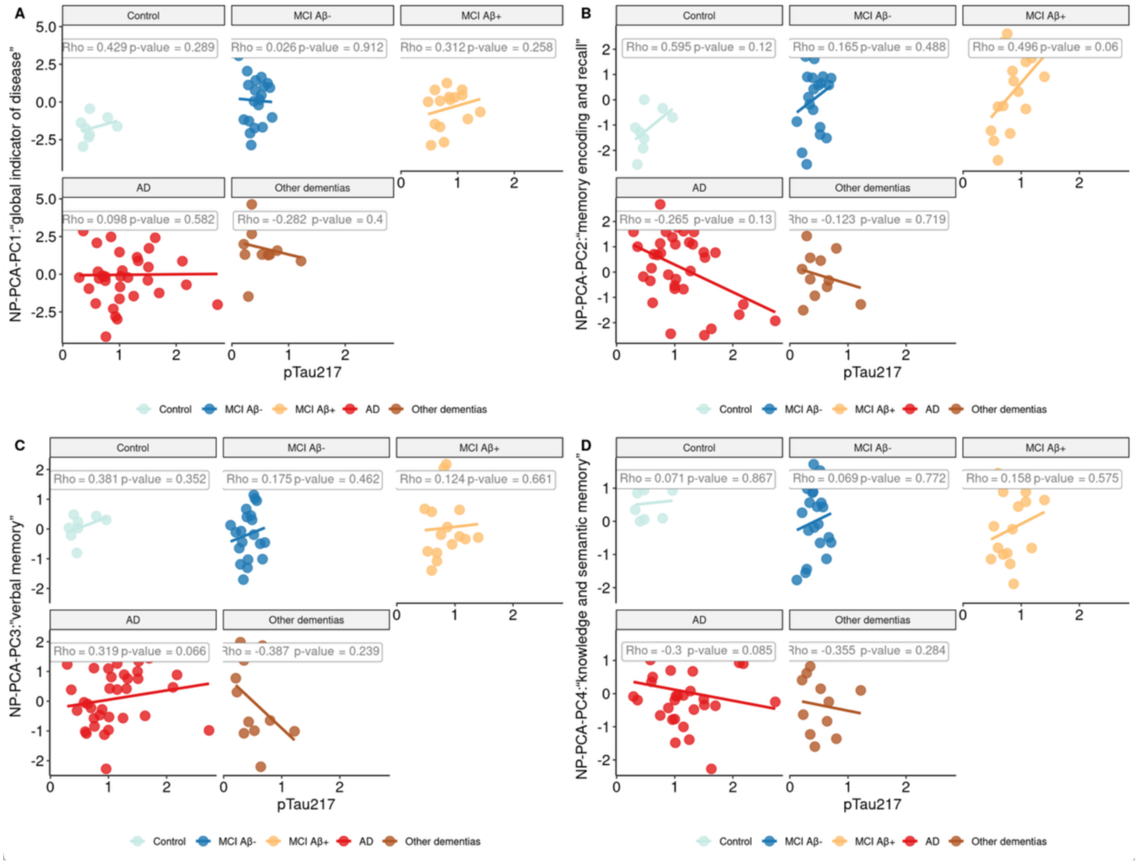

Plasma pTau217 vs Principal Components (PCs) from PCA in the diagnostic groups (Post Amy-PET diagnosis). Plasma pTau217 did not relate with any of the principal components identified by the PCA analysis in any of the groups. A tendency was observed in PC2 for the MCI Aβ+ group.

Supplementary Figure 3 – Plasma NFL vs RAVL learning (education adjusted) in controls

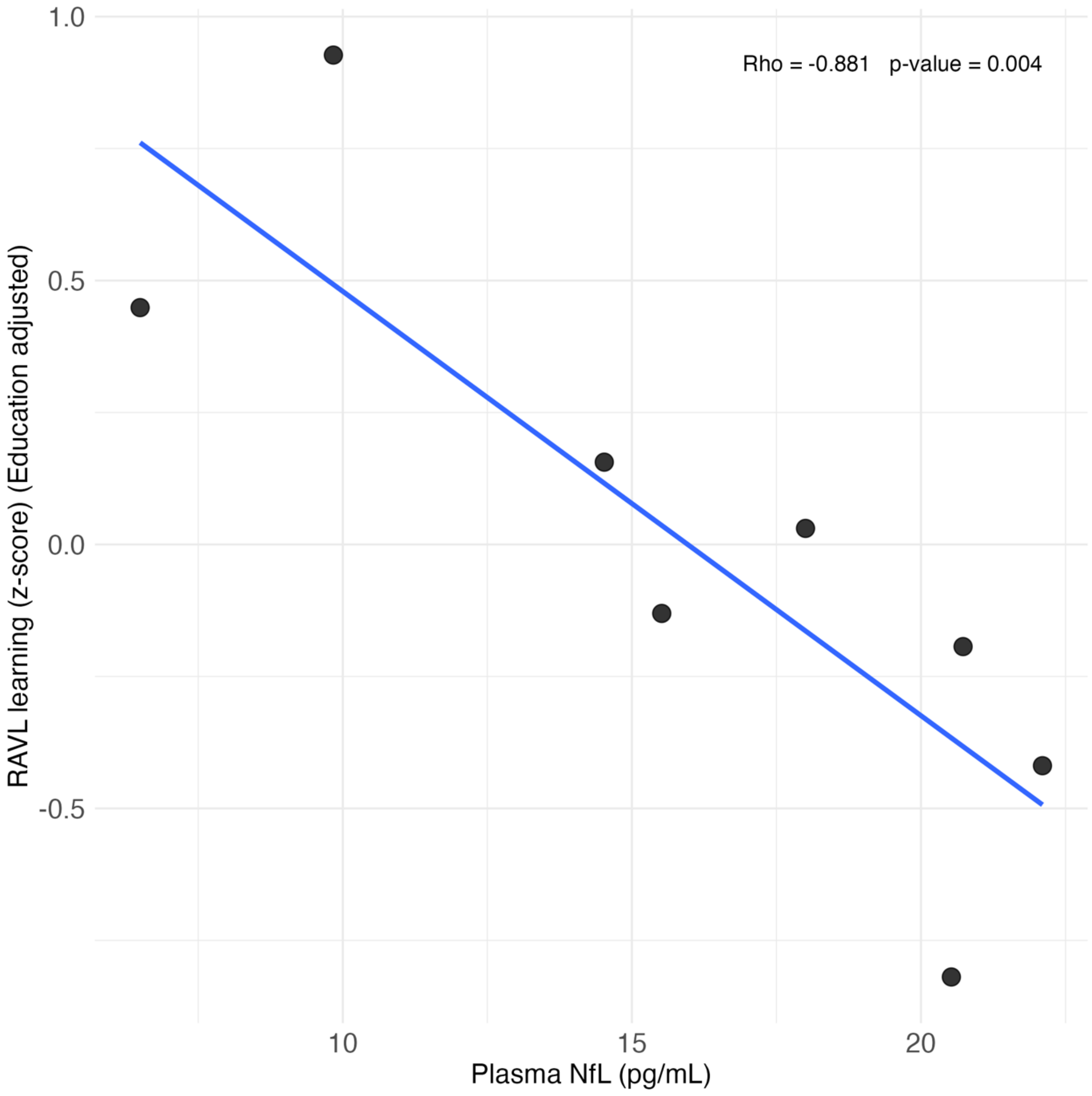

Supplementary Figure 4 – Calibration plots for LASSO prediction in both cohorts (A, whole cohort and B, MCI, Initial Diagnosis)

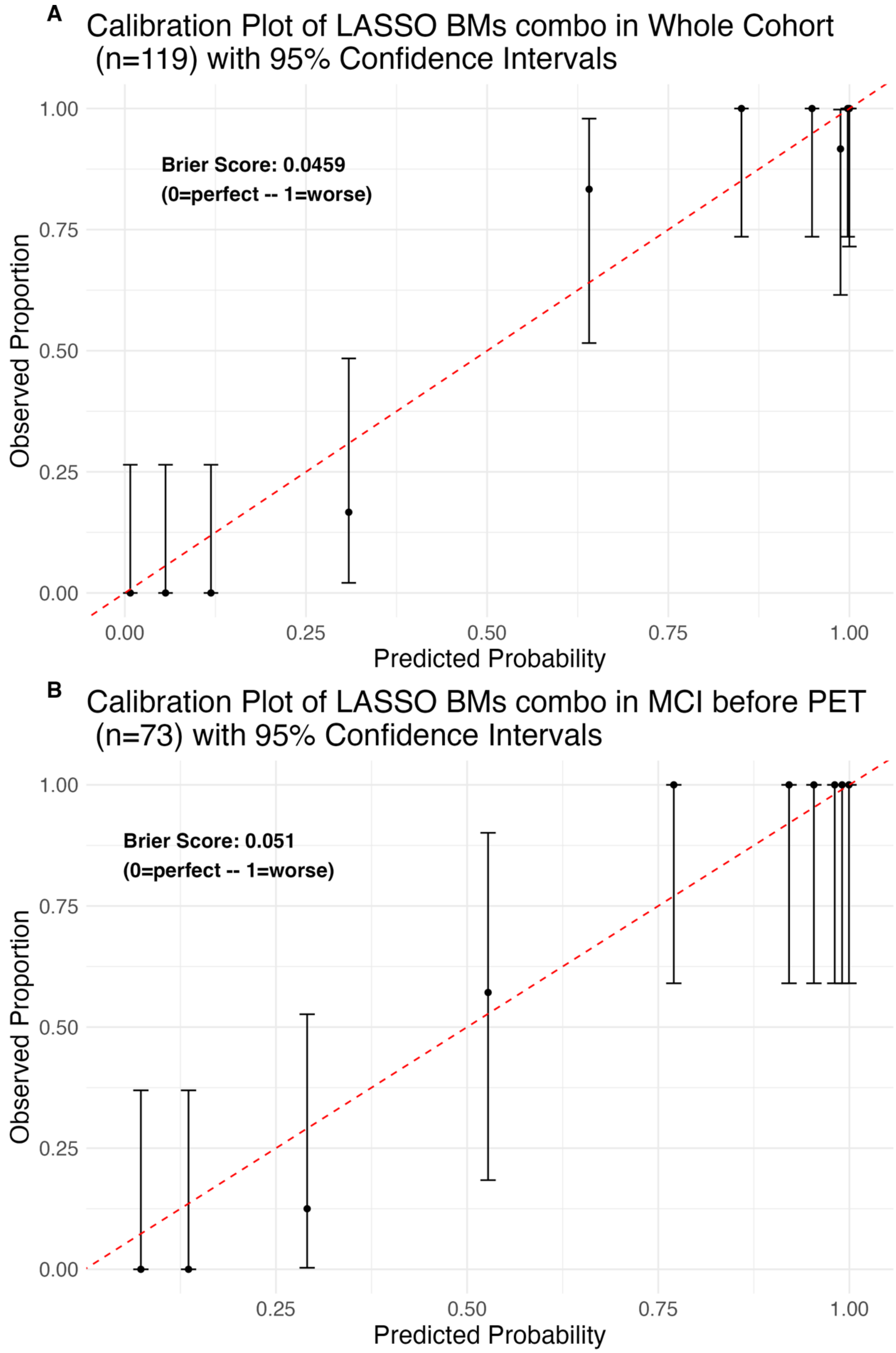

Calibration point confidence intervals overlap with perfect calibration line and Brier scores are close to 0 (perfect calibration)

Supplementary Figure 5 – Comparisons of tests across prevalences and cohorts (A,B,C vs D,E,F)

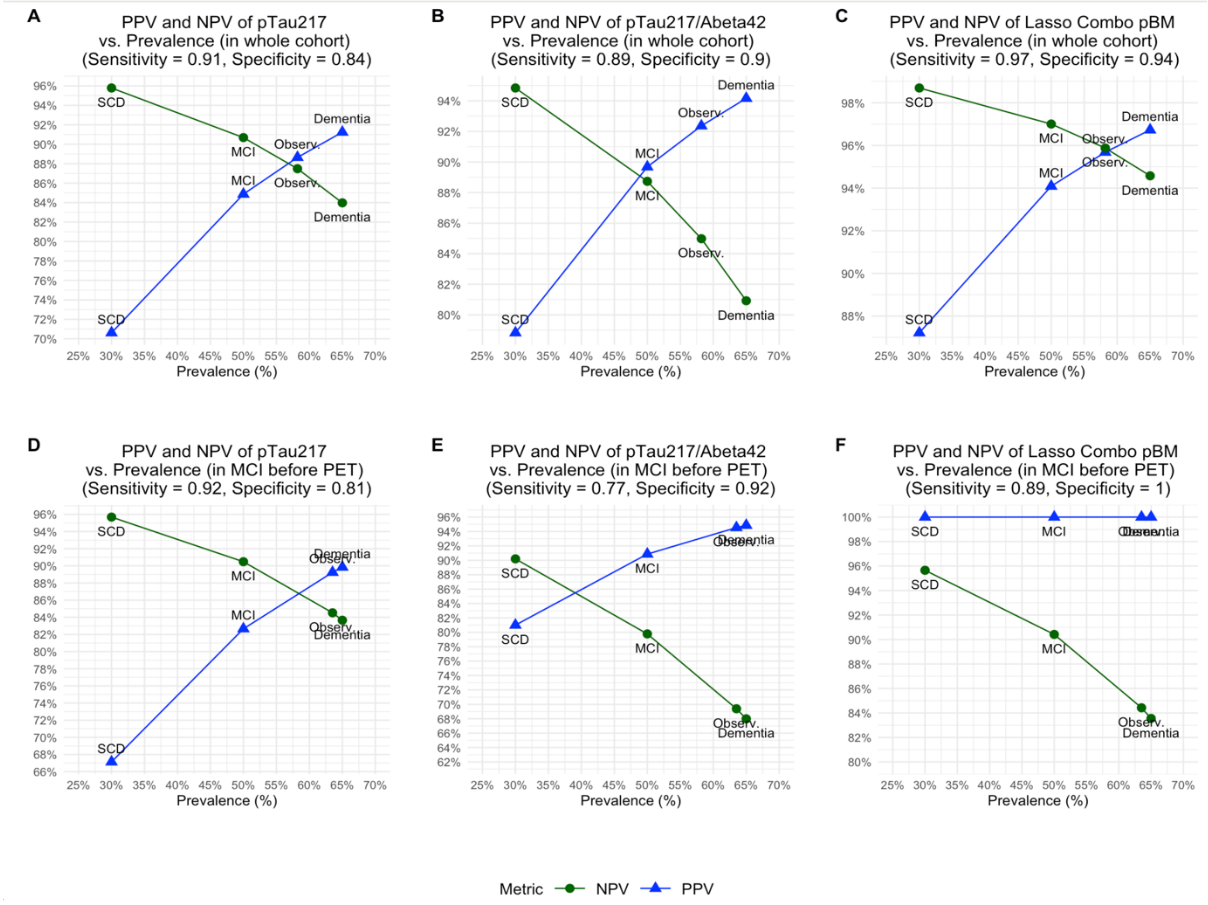

Statistical Analysis Results from Table 1

Summary of Overall Tests

Variable	Test	Statistic	DF	P_value
Age	Kruskal-Wallis (H statistic)	1.37	3	7.13e-01
Sex	Chi-Square (χ^2)	6.74	3	8.07e-02
Education	Kruskal-Wallis (H statistic)	2.67	3	4.45e-01
MMSE	Kruskal-Wallis (H statistic)	12.69	3	5.36e-03
Centiloid	Kruskal-Wallis (H statistic)	87.87	3	6.28e-19
Amyloid_Beta_VR_pos	Chi-Square (χ^2)	114.21	3	1.36e-24
Initial_diagnosis	Fisher (exact test)			1.00e-05

Post-hoc Comparisons: Dunn Test

Variable	Comparison	Statistic	P.adj
MMSE	AD - MCI Aβ-	-3.55e-01	7.23e-01
MMSE	AD - MCI Aβ+	-2.16	9.20e-02
MMSE	MCI Aβ- - MCI Aβ+	-1.69	1.09e-01
MMSE	AD - Other dementia	2.05	6.00e-02
MMSE	MCI Aβ- - Other dementia	2.15	6.30e-02
MMSE	MCI Aβ+ - Other dementia	3.54	2.42e-03
Centiloid	AD - MCI Aβ-	7.72	6.97e-14
Centiloid	AD - MCI Aβ+	1.45	1.76e-01
Centiloid	MCI Aβ- - MCI Aβ+	-4.85	2.44e-06
Centiloid	AD - Other dementia	7.18	2.14e-12
Centiloid	MCI Aβ- - Other dementia	-1.25e-01	9.01e-01
Centiloid	MCI Aβ+ - Other dementia	4.57	7.24e-06

Post-hoc Comparisons: Fisher Test

Variable	Comparison	P.adj
Amyloid_Beta_VR_pos	MCI Aβ- - MCI Aβ+	5.20e-12
Amyloid_Beta_VR_pos	MCI Aβ- - AD	1.15e-21
Amyloid_Beta_VR_pos	MCI Aβ- - Other dementia	4.42e-01
Amyloid_Beta_VR_pos	MCI Aβ+ - AD	3.26e-01
Amyloid_Beta_VR_pos	MCI Aβ+ - Other dementia	1.47e-09
Amyloid_Beta_VR_pos	AD - Other dementia	1.89e-17
Initial_diagnosis	MCI Aβ- - AD	3.55e-02
Initial_diagnosis	MCI Aβ- - Other dementia	2.50e-05
Initial_diagnosis	MCI Aβ+ - AD	1.86e-03
Initial_diagnosis	MCI Aβ+ - Other dementia	2.50e-05
Initial_diagnosis	AD - Other dementia	3.33e-05

Statistical Analysis Results from Fig 2

Summary of Overall Tests

Variable	Test	Statistic	DF	P_value
Centiloid	Kruskal-Wallis (H statistic)	87.87	3	6.28e-19
pTau217 (pg/mL)	Kruskal-Wallis (H statistic)	63.24	3	1.19e-13
GFAP (pg/mL)	Kruskal-Wallis (H statistic)	29.72	3	1.58e-06
pTau231 (pg/mL)	Kruskal-Wallis (H statistic)	12.52	3	5.81e-03
pTau181 (pg/mL)	Kruskal-Wallis (H statistic)	14.66	3	2.13e-03
NfL (pg/mL)	Kruskal-Wallis (H statistic)	2.92	3	4.04e-01
AB42 (pg/mL)	Kruskal-Wallis (H statistic)	12.36	3	6.25e-03
AB42/40 ratio	Kruskal-Wallis (H statistic)	7.60	3	5.50e-02
pTau217/AB42 ratio	Kruskal-Wallis (H statistic)	68.10	3	1.09e-14

Post-hoc Comparisons: Dunn Test

Variable	Comparison	Statistic	P.adj
Centiloid	MCI Aβ- - MCI Aβ+	4.85	2.44e-06
Centiloid	MCI Aβ- - AD	7.72	6.97e-14
Centiloid	MCI Aβ- - Other dementia	1.25e-01	9.01e-01
Centiloid	MCI Aβ+ - AD	1.45	1.76e-01
Centiloid	MCI Aβ+ - Other dementia	-4.57	7.24e-06
Centiloid	AD - Other dementia	-7.18	2.14e-12
pTau217 (pg/mL)	MCI Aβ- - MCI Aβ+	3.98	1.40e-04
pTau217 (pg/mL)	MCI Aβ- - AD	7.05	1.07e-11
pTau217 (pg/mL)	MCI Aβ- - Other dementia	7.35e-01	4.62e-01
pTau217 (pg/mL)	MCI Aβ+ - AD	1.75	9.59e-02

Variable	Comparison	Statistic	P.adj
pTau217 (pg/mL)	MCI Aβ+ - Other dementia	-3.08	3.06e-03
pTau217 (pg/mL)	AD - Other dementia	-5.62	5.73e-08
GFAP (pg/mL)	MCI Aβ- - MCI Aβ+	3.43	1.81e-03
GFAP (pg/mL)	MCI Aβ- - AD	5.16	1.48e-06
GFAP (pg/mL)	MCI Aβ- - Other dementia	1.69	1.09e-01
GFAP (pg/mL)	MCI Aβ+ - AD	6.97e-01	4.86e-01
GFAP (pg/mL)	MCI Aβ+ - Other dementia	-1.74	1.09e-01
GFAP (pg/mL)	AD - Other dementia	-2.90	7.53e-03
pTau231 (pg/mL)	MCI Aβ- - MCI Aβ+	8.49e-01	5.94e-01
pTau231 (pg/mL)	MCI Aβ- - AD	3.21	8.01e-03
pTau231 (pg/mL)	MCI Aβ- - Other dementia	5.65e-01	6.87e-01
pTau231 (pg/mL)	MCI Aβ+ - AD	1.87	1.22e-01
pTau231 (pg/mL)	MCI Aβ+ - Other dementia	-2.92e-01	7.70e-01
pTau231 (pg/mL)	AD - Other dementia	-2.33	5.93e-02
pTau181 (pg/mL)	MCI Aβ- - MCI Aβ+	1.05e-01	9.31e-01
pTau181 (pg/mL)	MCI Aβ- - AD	3.13	1.04e-02
pTau181 (pg/mL)	MCI Aβ- - Other dementia	2.07e-01	9.31e-01
pTau181 (pg/mL)	MCI Aβ+ - AD	2.60	1.88e-02
pTau181 (pg/mL)	MCI Aβ+ - Other dementia	8.70e-02	9.31e-01
pTau181 (pg/mL)	AD - Other dementia	-2.67	1.88e-02
NfL (pg/mL)	MCI Aβ- - MCI Aβ+	-3.10e-01	7.56e-01
NfL (pg/mL)	MCI Aβ- - AD	9.83e-01	4.88e-01
NfL (pg/mL)	MCI Aβ- - Other dementia	1.22	4.67e-01
NfL (pg/mL)	MCI Aβ+ - AD	1.19	4.67e-01
NfL (pg/mL)	MCI Aβ+ - Other dementia	1.40	4.67e-01
NfL (pg/mL)	AD - Other dementia	4.51e-01	7.56e-01
AB42 (pg/mL)	MCI Aβ- - MCI Aβ+	-4.92e-01	6.23e-01

Variable	Comparison	Statistic	P.adj
AB42 (pg/mL)	MCI Aβ- - AD	-1.62	1.59e-01
AB42 (pg/mL)	MCI Aβ- - Other dementia	1.78	1.51e-01
AB42 (pg/mL)	MCI Aβ+ - AD	-8.59e-01	4.68e-01
AB42 (pg/mL)	MCI Aβ+ - Other dementia	2.07	1.16e-01
AB42 (pg/mL)	AD - Other dementia	3.47	3.11e-03
AB42/40 ratio	MCI Aβ- - MCI Aβ+	-1.28e-01	8.98e-01
AB42/40 ratio	MCI Aβ- - AD	-1.38	3.02e-01
AB42/40 ratio	MCI Aβ- - Other dementia	1.28	3.02e-01
AB42/40 ratio	MCI Aβ+ - AD	-1.06	3.49e-01
AB42/40 ratio	MCI Aβ+ - Other dementia	1.28	3.02e-01
AB42/40 ratio	AD - Other dementia	2.71	4.09e-02
pTau217/AB42 ratio	MCI Aβ- - MCI Aβ+	3.55	6.56e-04
pTau217/AB42 ratio	MCI Aβ- - AD	6.88	3.64e-11
pTau217/AB42 ratio	MCI Aβ- - Other dementia	-1.87e-01	8.52e-01
pTau217/AB42 ratio	MCI Aβ+ - AD	2.07	4.65e-02
pTau217/AB42 ratio	MCI Aβ+ - Other dementia	-3.52	6.56e-04
pTau217/AB42 ratio	AD - Other dementia	-6.48	2.72e-10

Statistical Analysis Results from Figure 7

Summary of Overall Tests

Variable	Test	Statistic	DF	P_value
NP__PC1__unadj	Kruskal-Wallis (H statistic)	20.51	4	3.95e-04
NP__PC2__unadj	Kruskal-Wallis (H statistic)	8.61	4	7.16e-02
NP__PC3__unadj	Kruskal-Wallis (H statistic)	1.37	4	8.49e-01
NP__PC4__unadj	Kruskal-Wallis (H statistic)	6.15	4	1.88e-01

Post-hoc Comparisons: Dunn Test

Variable	Comparison	Statistic	P.unadj	P.adj
NP__PC1__unadj	Control - MCI Aβ-	2.73	6.25e-03	1.56e-02
NP__PC1__unadj	Control - MCI Aβ+	1.93	5.32e-02	7.60e-02
NP__PC1__unadj	Control - AD	2.65	8.14e-03	1.63e-02
NP__PC1__unadj	Control - Other dementia	4.40	1.07e-05	1.07e-04
NP__PC1__unadj	MCI Aβ- - MCI Aβ+	-8.71e-01	3.84e-01	4.80e-01
NP__PC1__unadj	MCI Aβ- - AD	-3.69e-01	7.12e-01	7.12e-01
NP__PC1__unadj	MCI Aβ- - Other dementia	2.40	1.63e-02	2.72e-02
NP__PC1__unadj	MCI Aβ+ - AD	6.24e-01	5.33e-01	5.92e-01
NP__PC1__unadj	MCI Aβ+ - Other dementia	3.02	2.52e-03	1.25e-02
NP__PC1__unadj	AD - Other dementia	2.90	3.74e-03	1.25e-02
NP__PC2__unadj	Control - MCI Aβ-	2.34	1.93e-02	6.44e-02
NP__PC2__unadj	Control - MCI Aβ+	2.53	1.15e-02	5.77e-02
NP__PC2__unadj	Control - AD	2.78	5.41e-03	5.41e-02
NP__PC2__unadj	Control - Other dementia	1.66	9.69e-02	2.42e-01
NP__PC2__unadj	MCI Aβ- - MCI Aβ+	3.72e-01	7.10e-01	7.88e-01
NP__PC2__unadj	MCI Aβ- - AD	4.06e-01	6.84e-01	7.88e-01
NP__PC2__unadj	MCI Aβ- - Other dementia	-5.52e-01	5.81e-01	7.88e-01

Variable	Comparison	Statistic	P.unadj	P.adj
NP__PC2__unadj	MCI Aβ+ - AD	-4.09e-02	9.67e-01	9.67e-01
NP__PC2__unadj	MCI Aβ+ - Other dementia	-8.43e-01	3.99e-01	6.66e-01
NP__PC2__unadj	AD - Other dementia	-9.28e-01	3.54e-01	6.66e-01
NP__PC3__unadj	Control - MCI Aβ-	-7.25e-01	4.68e-01	9.89e-01
NP__PC3__unadj	Control - MCI Aβ+	-3.89e-01	6.97e-01	9.89e-01
NP__PC3__unadj	Control - AD	-1.32e-02	9.89e-01	9.89e-01
NP__PC3__unadj	Control - Other dementia	-4.69e-01	6.39e-01	9.89e-01
NP__PC3__unadj	MCI Aβ- - MCI Aβ+	3.90e-01	6.97e-01	9.89e-01
NP__PC3__unadj	MCI Aβ- - AD	1.06	2.90e-01	9.89e-01
NP__PC3__unadj	MCI Aβ- - Other dementia	2.28e-01	8.20e-01	9.89e-01
NP__PC3__unadj	MCI Aβ+ - AD	5.33e-01	5.94e-01	9.89e-01
NP__PC3__unadj	MCI Aβ+ - Other dementia	-1.20e-01	9.04e-01	9.89e-01
NP__PC3__unadj	AD - Other dementia	-6.13e-01	5.40e-01	9.89e-01
NP__PC4__unadj	Control - MCI Aβ-	-1.55	1.21e-01	3.51e-01
NP__PC4__unadj	Control - MCI Aβ+	-2.02	4.37e-02	2.19e-01
NP__PC4__unadj	Control - AD	-1.35	1.76e-01	3.51e-01
NP__PC4__unadj	Control - Other dementia	-2.20	2.80e-02	2.19e-01
NP__PC4__unadj	MCI Aβ- - MCI Aβ+	-6.86e-01	4.93e-01	6.16e-01
NP__PC4__unadj	MCI Aβ- - AD	4.14e-01	6.79e-01	7.28e-01
NP__PC4__unadj	MCI Aβ- - Other dementia	-9.91e-01	3.22e-01	4.59e-01
NP__PC4__unadj	MCI Aβ+ - AD	1.13	2.58e-01	4.30e-01
NP__PC4__unadj	MCI Aβ+ - Other dementia	-3.47e-01	7.28e-01	7.28e-01
NP__PC4__unadj	AD - Other dementia	-1.41	1.59e-01	3.51e-01
